# Curation of Mass Spectrometry Reference Data for Improved Identification and Dereplication of Cyanobacterial Specialized Metabolites

**DOI:** 10.1021/acs.jnatprod.6c00107

**Published:** 2026-04-23

**Authors:** Franziska Schanbacher, Anne Dax, Michael A. Stravs, Timo H. J. Niedermeyer, Elisabeth M.-L. Janssen

**Affiliations:** † Department of Pharmaceutical Biology, Institute of Pharmacy, Freie Universität Berlin, 14195 Berlin, Germany; ‡ 28499 Eawag, Swiss Federal Institute of Aquatic Science and Technology, 8600 Dübendorf, Switzerland

## Abstract

High-resolution tandem mass spectrometry (HRMS/MS) is a powerful tool for screening organic compounds in complex samples. A critical step in identifying candidate structures is the comparison of sample HRMS/MS spectra with those of reference spectral libraries. The effectiveness of this spectral matching hinges on two key factors: (i) how well the library’s content aligns with the suspect compound list and (ii) the quality and diversity of the reference spectra for each compound. Yet the scarcity of natural product reference materials on the market often necessitates non-targeted analysis. In this study, we systematically acquired and curated HRMS/MS reference spectra for specialized metabolites from cyanobacteria, which are vastly underrepresented in the current libraries. Previously, MassBank EU included spectra for only 14 such compounds. We have significantly expanded the publicly available data, contributing 2905 unique spectra representing 150 distinct cyanobacterial metabolites. A proof-of-concept analysis demonstrates up to 5-fold increased annotation success and revealed shortcomings in current libraries, underscoring the need for continued data enrichment. In particular, future efforts should prioritize the inclusion of HRMS/MS spectra for diverse adduct ions to improve identification confidence and broaden the analytical coverage.

## Graphical abstract

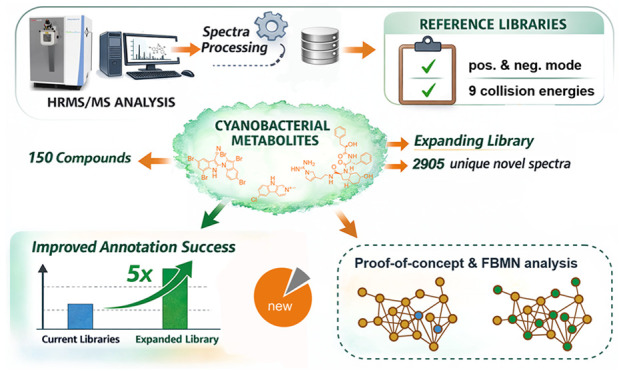

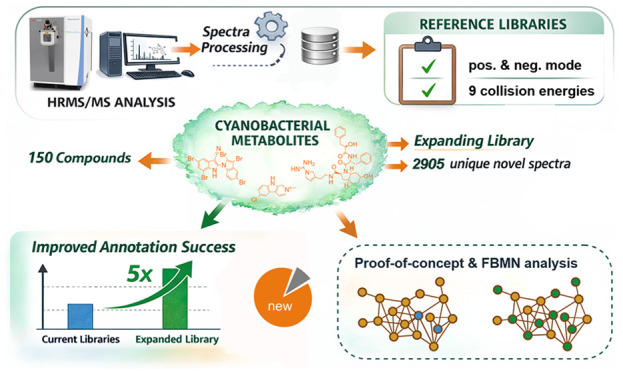

Mass spectrometry (MS), especially high-resolution tandem MS (HRMS/MS), is widely used to screen samples or sample sets for organic compounds, including metabolites, with the example of specialized metabolites (SMs) from cyanobacteria focused on herein. Moreover, HRMS/MS-based screening approaches have become an integral component of rapid early stage dereplication workflows, minimizing the risk of rediscovering known compounds in natural product research.^1–3^ Common workflows also incorporate efficient annotation strategies of known compounds to support the comprehensive structure elucidation of yet undescribed congeners.^4^

The two main workflows in mass spectrometry analysis are target analysis and non-target analysis.^5^ Target analysis focuses on compounds for which laboratory methods, including instrument settings, are known and reference standards are available to the analyzing laboratory. While reference standards, including isotopically labeled ones, are commercially available for most micropollutants such as pharmaceuticals, pesticides, and industrial chemicals, standards for specialized metabolites are scarce and often expensive when derived from purified raw materials rather than chemical synthesis. Consequently, the target analysis of specialized metabolites is often limited. When reference materials and prior knowledge of optimal instrument methods are unavailable, non-target analysis can be applied. Suspect screening is a type of non-target analysis that searches MS data against a suspect list of compounds whose molecular formulas and chemical structures are known from the literature. Suspect screening enables researchers to expand beyond the limited number of compounds available for targeted analysis and to increase the likelihood of identifying suspects of interest across a wide chemical space in a given sample. The confidence of the identity of a compound is often given in a level system following recommendations by Schymanski et al.:^6^ Level 1 for confirmed structures from reference material purity (>95%) with previous MS^2^ annotation; Level 2 for probable structures, where Level 2a is assigned when a reference MS^2^ spectrum can be matched and Level 2b is assigned when comprehensive diagnostic evidence of MS^2^ fragments and experimental context elute to the proposed structure; and Level 3 for tentative candidates when evidence of MS^2^ fragments exists but is insufficient to assign one exact structure (e.g., the case of isomers, or incomplete fragmentation information). The suspect list has to be defined by the analyzing laboratory and can range up to large and chemically complex lists such as the chemical space provided by PubChem (118 million unique chemical structures, 2025). However, their size and heterogeneity limit screening workflows, so more focused lists are oftentimes preferable. More compact but still broadly applicable resources, such as PubChemLite (566,122 compounds, 2025),^7^ can offer a more manageable alternative, while even more specific focal areas may be selected, for example, bacterial metabolites in The Natural Products Atlas (36,545 compounds, 2024).^8,9^ More defined lists based on disciplinary interests can offer highly curated, often manually verified compound information. For example, SMs from cyanobacteria in CyanoMetDB contain 3084 compounds (version 03 from 2024) with primary references and structural codes (e.g., SMILES).^10,11^ Cyanobacteria can be prolific sources of relevant SMs, and improvements in dereplication will enhance their identification for environmental monitoring and drug-discovery applications. Even defined lists like CyanoMetDB consist of compounds covering a diverse chemical space, and a compromise has to be made regarding the instrument methods selected for the analysis of samples. In suspect screening, while it is not known *a priori* which compounds can be expected in the sample of interest, a suspicion might exist, for example, that the sample contains cyanobacteria and thus some of their SMs are suspected to be present. Hence, the respective suspect list is considered while designing the instrument method, e.g., to define the scan range setting and to include a defined target mass list to trigger the fragmentation spectra of those suspects. In contrast to target analysis, the instrument settings will not be optimized for each analyte and rather present a compromise that allows as many compounds from the suspect list to be covered as possible, including a selection of conditions for fragmentation (e.g., collision energies). These compromise parameters allow the acquisition of HRMS spectra across a wide range of features and are designed to enable downstream dereplication or tentative annotation, even though the measurement conditions are not individually optimized for each compound.

Following data acquisition, the data analysis workflow of suspect screening broadly comprises two steps. First, the MS features in the sample are compared to the suspect list to identify tentative candidates whose precursor specifications, molecular formula, mass error, and isotope pattern of the MS^1^ scans match. In a second and vital step, a higher confidence of the identity of candidate structures is achieved by matching the measured MS^2^ fragmentation spectra with spectra in a spectral reference library (Level 2).^6^ No prior knowledge of the fragmentation of the probable structure needs to be known at this stage. Computational tools such as NIST MS search^12^ and The Global Natural Products Social Molecular Networking platform (GNPS, http://gnps.ucsd.edu)^13^ can facilitate automated matching of measured spectra with reference spectra. Further, platforms such as SIRIUS^14–18^ provide both reference spectra search and advanced *in-silico* methods, which include molecular formula annotation, structure database searches for compounds without entries in spectral libraries (i.e., in addition to spectral database searches), and compound class prediction, which can provide additional information in non-target analyses, suspect screening, and dereplication approaches.

Spectral matching is a cornerstone of successful suspect screening. Its success depends on (i) the compatibility of available compounds in a reference spectral library with the suspect list of interest and (ii) the quality and diversity of the deposited reference spectra for a given compound.^19,20^ Examples of widely used reference spectra libraries are MassBank EU, GNPS, and the National Institute of Standards and Technology (NIST) reference library. MassBank EU has been available since 2011 as one of the first publicly available mass spectral libraries. Since, it developed into a truly open access, open data, open-source resource with highly curated spectra to perform suspect screening.^21,22^ MassBank is integrated in other resources such as PubChem, the US EPA CompTox Dashboard, the NORMAN Database System, and RforMassSpectrometry. As of 2025, Massbank EU offers references spectra for 18,529 unique compounds and 119,845 unique spectra that are publicly available.^23^ NIST, which is only commercially available, offers spectra for 51,501 compounds and 2.4 million spectra.^24^ GNPS contains an open-access knowledge base for community-wide sharing of MS data as reference spectra (GNPS-Collections), including also external data, such as MassBank EU, as well as a crowd-sourced library of community-contributed spectra with a lower level of curation (GNPS-Community). Reference spectra have so far been limited to a subset of compounds: (i) those for which commercially available reference materials exist and (ii) the few spectra derived from isolated or otherwise annotated compounds, rather than from commercially available standards. The limitation of available reference materials for natural products is a major challenge, resulting in the lack of representation in spectral libraries. Before the addition of new spectra outlined herein, cyanobacterial SMs available in MassBank EU comprised only 14 compounds, including eight microcystin congeners (microcystin-LR, -LA, -LF, -LY, -LW, -RR, -YR, [dAsp,^3^ Dhb,^7^]-microcystin-RR) as well as nodularin-R, aerucyclamide A, oscillamide Y, and anabaenopeptins A, B, and NZ857. These entries comprise a total of 215 unique spectra, for [M + H]^+^ and [M – H]^−^ precursor ions with a range of normalized collision energies (15–180%, NCE) and MS^2^ resolution ranging from 7,500 to 35,000 (Table S1).

Here, we systematically acquired and processed reference spectra of cyanobacterial SMs, not only from reference materials but also mainly from semipurified or crude biomass extracts, to enable suspect screening via spectral library matching. We followed established procedures for measurement and data processing and included compounds amenable to reversed-phase liquid chromatography. Detection was performed using HRMS/MS with electrospray ionization (ESI Orbitrap). Instrument settings covered typical non-target screening conditions, including positive and negative ionization modes and a wide range of collision energies. Raw data were processed with the open-source R package RMassBank (https://github.com/meowcat/RMassBank-scripts) for automated recalibration, cleanup, intensity correction, metadata retrieval, and MassBank export.^25^ In total, 2905 unique spectra of 150 SMs were uploaded to MassBank EU. A proof-of-concept study identifies missing compound families or representatives.

## Results and Discussion

### Acquisition and Processing of Reference Spectra

In total, 411 unique compounds were analyzed. Depending on the chromatographic peak width, 200–500 individual spectra were recorded per compound and spread over 36 spectra types (9 collision energies, 2 ionization modes, and 2 HRMS^2^ scan modes, (Figure 6)) with 5–15 replicates for each spectrum type. Of all analyzed samples, 365 compounds were well detected, 277 compounds passed the data processing, and 150 compounds passed a final manual check by the expert who supplied the material and was familiar with the respective expected tandem mass spectrum (Figure 1A). A total of 2905 unique new spectra of these 150 compounds could be uploaded to MassBank EU. An overview is provided in Table S4. The main reasons why compounds were rejected during data processing were insufficient intensity of the precursor peak and/or too many interferences from the background. When intensity issues occurred, higher injection volumes, respectively concentration of the samples was tested where possible. As this solution also introduced more matrix to the instrument, an additional cleanup step (e.g., solid phase extraction) or higher concentrated biomass available for extraction would be necessary to obtain sufficient precursor intensities. To combat the issue of interferences in those samples where only crude extracts were available, a cleanup step is also required in the future. While we have reanalyzed 54 compounds with adopted chromatography, even more nuanced chromatographic settings will improve precursors detection and separation from isobars for a wider range of SMs (e.g., HILIC, ion mobility etc.)

**Figure 1:**
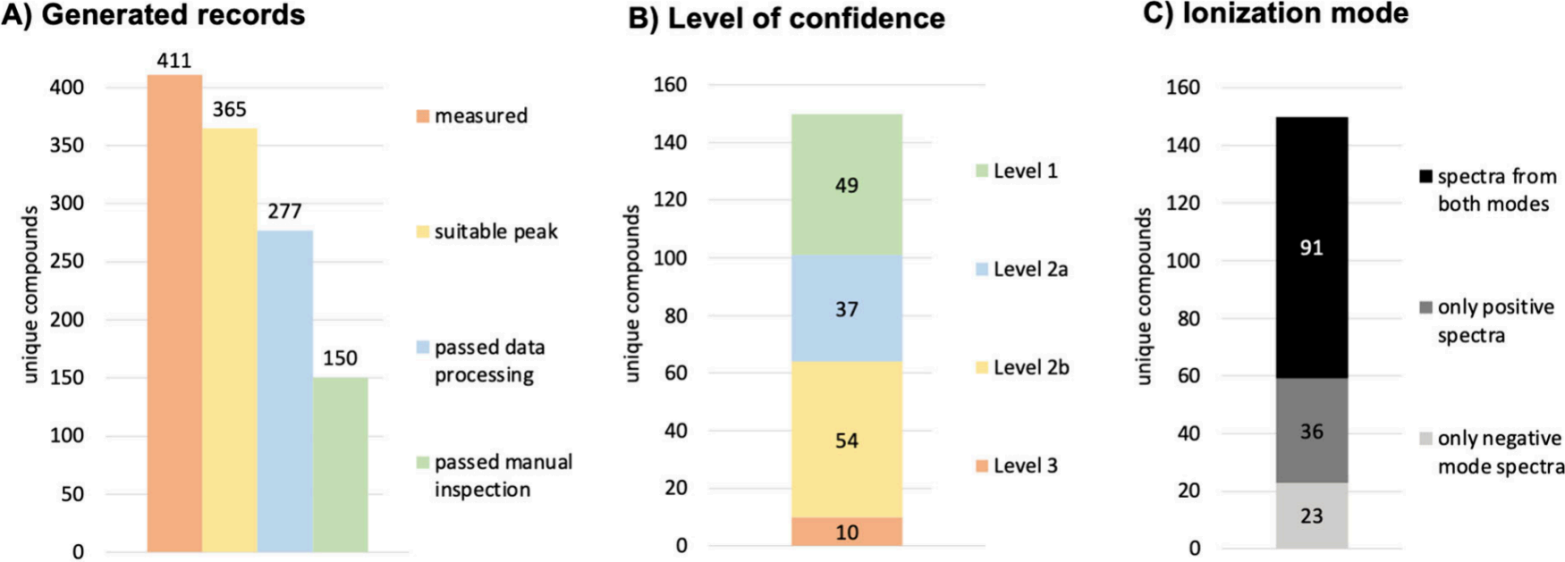
**Overview of results.** (A) 411 unique compounds were analyzed, 365 compounds were detected well, 277 compounds passed data processing, and 150 compounds passed manual inspection, were approved for record generation, and uploaded to MassBank EU. (B) Level of confidence reached for approved records ranges from 49 compounds on Level 1 to 37/54 compounds of Level 2a/2b and 10 compounds on Level 3. (C) Availability of ionization mode in spectra types showed both modes for 91 compounds, only positive mode for 36 compounds, and only negative mode for 23 compounds.

Despite many spectra not passing quality control, we significantly increased the coverage of SMs from cyanobacteria in MassBank EU, notably expanding the chemical space. We provided new spectra for 11 compounds that were previously represented in MassBank EU (marked with # in Table S4), and introduced 140 compounds as novel entries into the spectral library. Spectra for anabaenopeptin NZ 857 were not recorded again herein, as we previously uploaded the spectra from the bioreagent. For example, 21 microcystin congeners were added to the existing eight. Many new compound classes are represented for the first time, including cyanopeptolins, microginins, aeruginosins, and spumigins, among many others. Overall, we increased the number of unique spectra of SMs from cyanobacteria from 215 to 2905 with higher resolution and a wider range of collision energies.

Of the 150 unique compounds, 49 compounds were identified at confidence Level 1 (reference materials). For 17 of those Level 1 compounds, a crude extract had to be used for recording the spectra herein, because the previous reference material was not available anymore, yet the certainty of compound identity remains at Level 1 (see also extended Table S4). In addition, 37 compounds at confidence Level 2a, 54 compounds at confidence Level 2b, and 10 compounds at confidence Level 3 (Figure 1B). The confidence level is marked in the MassBank EU record for each compound. All records derived from non-reference materials (lower than Level 1) are marked with “TENTATIVE” in the record title which has to be taken into account when library matches are achieved during data processing. Care should be taken with confidence Level 3 library matches, since the annotation of the compound identity was partially inconclusive regarding other structural isomers and the same MassBank EU record was associated with all isomer names in the database (see last 10 entries in Table S4). Note that these records are not counted multiple times when referring to unique compounds herein. Regarding the ionization mode, 23 compounds could only be detected in negative mode, 36 could only be detected in positive mode, and 91 compounds were detected in both ionization modes (Figure 1C).

### Proof-of-Concept Study

In a proof-of-concept study, the impact of the newly deposited HRMS^2^ reference spectra on the annotation of compounds known by the authors to be present in specific cyanobacterial extracts was investigated (Table S2). Annotation rates were compared before and after the addition of the reference data discussed above, demonstrating how the expanded spectral library influences the identification of cyanobacterial SMs. Recovery rates were assessed by determining whether these newly added reference compounds, several of which are present in the analyzed extracts (Table S2), could be rediscovered and, if so, the degree of spectral similarity between the reference spectra and the measured HRMS^2^ spectra. In total, feature-based molecular networking (FBMN) of the positive-mode data of the selected strains revealed 3351 nodes, of which 2233 features were organized into clusters. Of these, 26 nodes were annotated via spectral-library matching, while 139 were annotated through structure-database searches for a total of 165 annotated nodes. Notably, 16 of the 26 spectral-library-matching annotations — and thus 155 of the 165 annotations overall (structure-database searches + spectral-library matching) — could be achieved exclusively through the newly recorded reference spectra generated in this study (Table S5). The remaining 10 spectral-library matching annotations were based on spectra already present in the previous MassBank data set. Of these, six were annotated exclusively through MassBank data via spectral-library matching in SIRIUS, whereas the other four could likewise be annotated using the corresponding user-contributed cyanobacterial spectra available on the GNPS platform. Moreover, one annotation (microginin FR1, Figure 2D) obtained through the structure-database search was confirmed using a user-contributed spectrum from the GNPS platform. Furthermore, the newly added reference data enabled the identification of five compound-family clusters that had previously remained undetected (Figure 2, C–F). Using the annotated SMs as key nodes in the FBMN analysis, the confidence of structure database–based annotations for the corresponding congeners could be improved (Figure 2, A–F). The inclusion of the new reference spectra led to a substantial increase in the overall annotation rate from roughly 0.3% to 0.8%, a 2.5-fold increase. When cluster-based annotation relationships are considered, such as in the cyanobacterin cluster or the cryptophycin cluster (Figure 2C and E), the total annotation coverage across the data set increases to approximately 1.6%. This is particularly evident for the cyanobacterin cluster (Figure 2C), where cluster-based annotation enabled the manual postannotation of additional congeners of cyanobacterin and anhydrocyanobacterin. The compounds had previously been reported in the literature but were not represented in public databases such as CyanoMetDB, which at the time of our study lacked both spectral data and structural entries (e.g., SMILES), and therefore annotation of these compounds was not accessible through structure database searches or spectral library matching. Seven additional annotations based on the newly recorded reference spectra were enabled through FBMN analysis of the negative-mode data (Figure 3).

**Figure 2:**
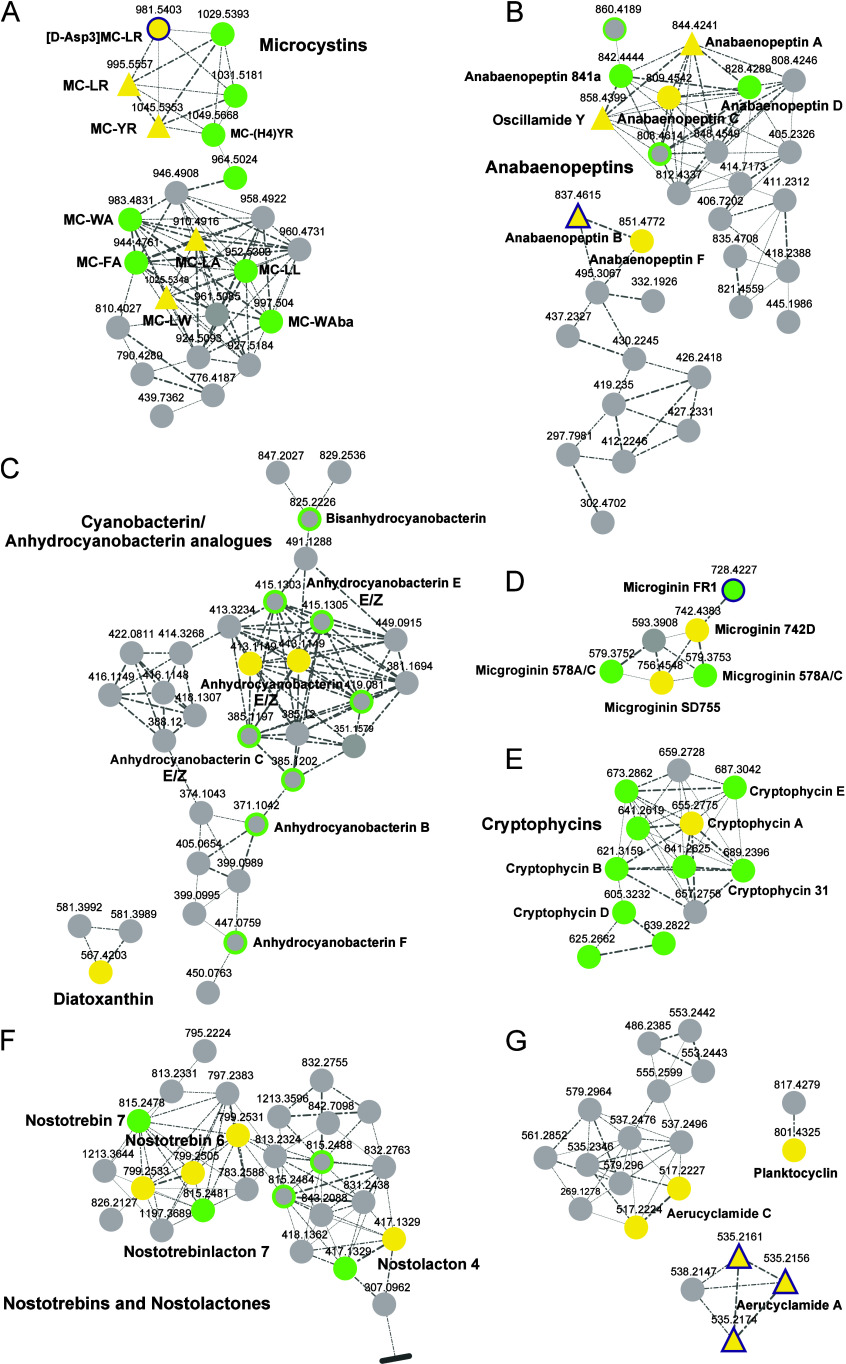
Feature Based Molecular Networking (FBMN) analysis (subsection, only annotated clusters of the molecular network are shown) of a sample set of cyanobacterial extracts with major compounds known *a priori* to the authors (Table S2) for chemical space visualization: positive mode data. Compound annotation was achieved through spectral library matching and structure database search using the SIRIUS platform. Nodes are connected for cosine scores ≥0.7, with edge width correlating to cosine scores. The area of clusters with features annotated via spectral library matching is highlighted. Annotations obtained through spectral library matching are highlighted in yellow, and those derived from structure database searches are shown in green. Triangular nodes indicate spectra previously included in the MassBank database, while nodes outlined in purple represent features additionally annotated via GNPS. Features without annotation, either corresponding to cyanobacterial SMs or uncharacterized compounds in general, are shown in gray. To enhance readability, single unconnected nodes were excluded from the FBMN network representation. Clusters of (A) microcystins, (B) anabaenopeptins, (C) cyanobacterins,^26^ (D) microginins, (E) cryptophycins, (F) nostotrebins/nostolactons (the cluster is not shown in its entirety due to space constraints; no annotations were associated with the omitted features), and (G) aeruclycamides, where spectral annotation of main compounds was enabled by newly acquired HRMS^2^ spectra recorded in this study, demonstrating enhanced annotation coverage.

**Figure 3:**
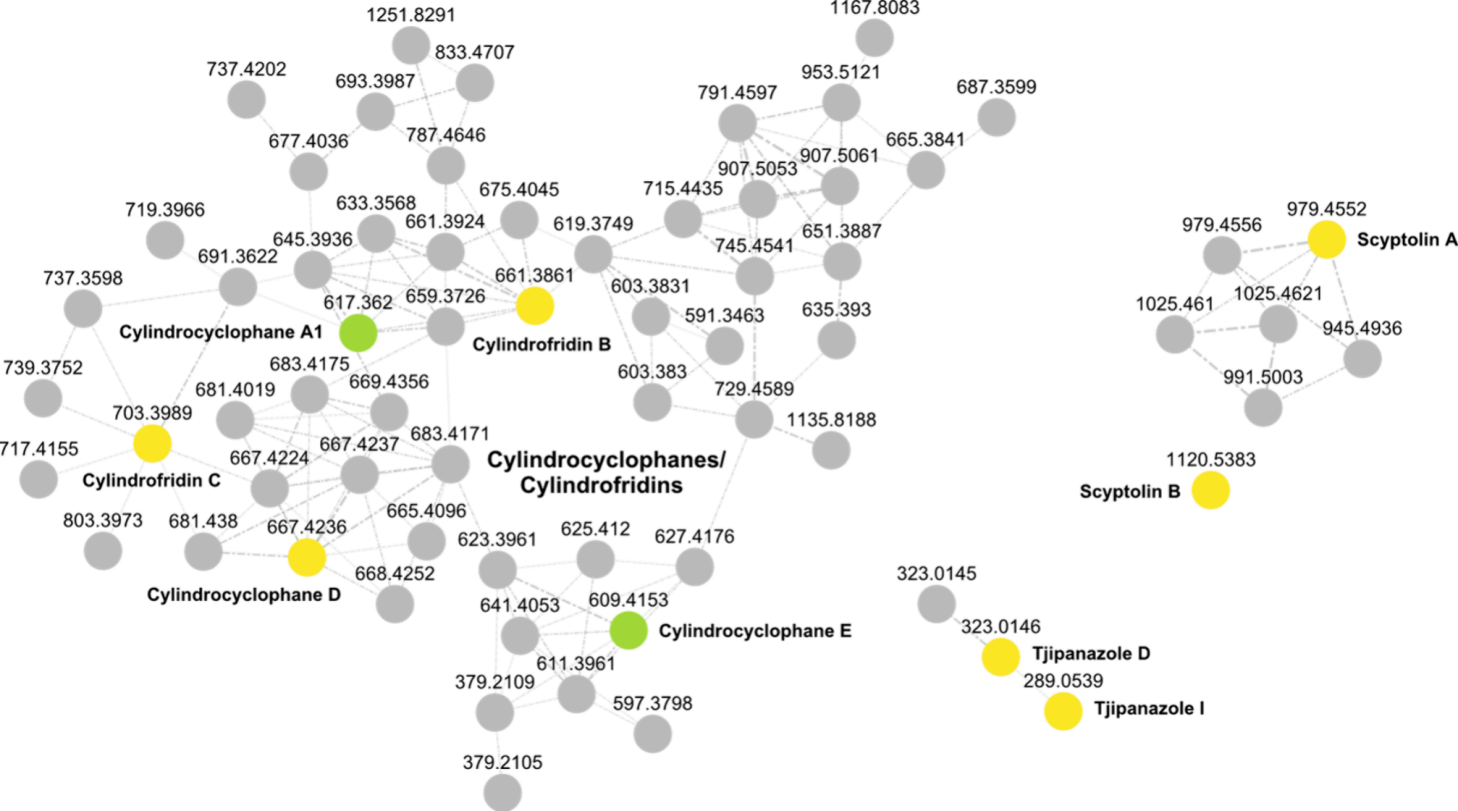
Feature Based Molecular Networking (FBMN) analysis (selected clusters) of a sample set of cyanobacterial extracts with major compounds known *a priori* to the authors (Table S2): negative ionization mode data. Compound annotation was achieved through spectral library matching and structure database search using the SIRIUS platform. The clusters corresponding to cylindrocyclophanes/cylindrofridins, scyptolins, and tjipanazoles were identified exclusively in negative ionization mode data. Nodes are connected for cosine scores ≥0.7, with edge width correlating to cosine scores. Annotations obtained through spectral library matching are highlighted in yellow, and those derived from structure database searches are shown in green. Features without annotation, either corresponding to cyanobacterial SMs or uncharacterized compounds in general, are shown in gray.

Except for one compound, all expected SMs were successfully rediscovered (Table S5). Although reference spectra for acutiphycin were acquired, no corresponding features were identified in the extracts. The pure substance was used for reference spectrum acquisition, and inclusion lists were generated based on [M + H]^+^ or [M – H]^−^ ions. However, acutiphycin tends to form adducts and undergoes water loss during ionization. In some cases, the [M + H]^+^ signal was very low, resulting in insufficient ion intensity for MS^2^ fragmentation under the applied method. Furthermore, the HRMS^2^ spectra differ between the [M + H]^+^, [M + Na]^+^, and [M – H_2_O]^+^ ions. This example highlights the necessity of accounting for potential adduct formation when reference spectra are acquired in future studies.

The analysis also revealed the remaining gaps in the reference data, pinpointing which representative members of each compound family should be prioritized for future measurements. For example, the microginin cluster highlights the need for additional reference data. In untargeted screening, acquisition parameters always involve compromise and are not optimized for specific compound classes. Except for microginin SD755 and microginin 742D, no other microginin congeners could be annotated from spectral data (Figure 2D), and their matching scores are low (Table S5, 32% and 24%). In addition to the two annotated microginins, other microginins are expected to be present. Although spectral annotation for these additional compounds was not possible, the low mass deviations between exact and accurate masses, together with their coclustering in the FBMN with the annotated microginins, suggest that these features likely correspond to the microginins of interest. These findings highlight not only the necessity of optimizing acquisition parameters to reliably detect and annotate microginins but also the importance of systematically expanding reference data. As illustrated in Figure 4, the lack of reference spectra affects not only microginins but also other relevant cyanobacterial peptides.^27^ The clusters shown in that figure correspond to members of several compound families for which spectral references are still unavailable, highlighting the demand for additional data to enable more confident annotation across diverse compound classes, e.g., the large compound family of hapalindoles, fischerindoles, and welwitindolinones^28–33^ as well as micropeptins,^34^ cyanopeptolins^35^ (see also Figure 5C), or aeruginosins.^36^

**Figure 4:**
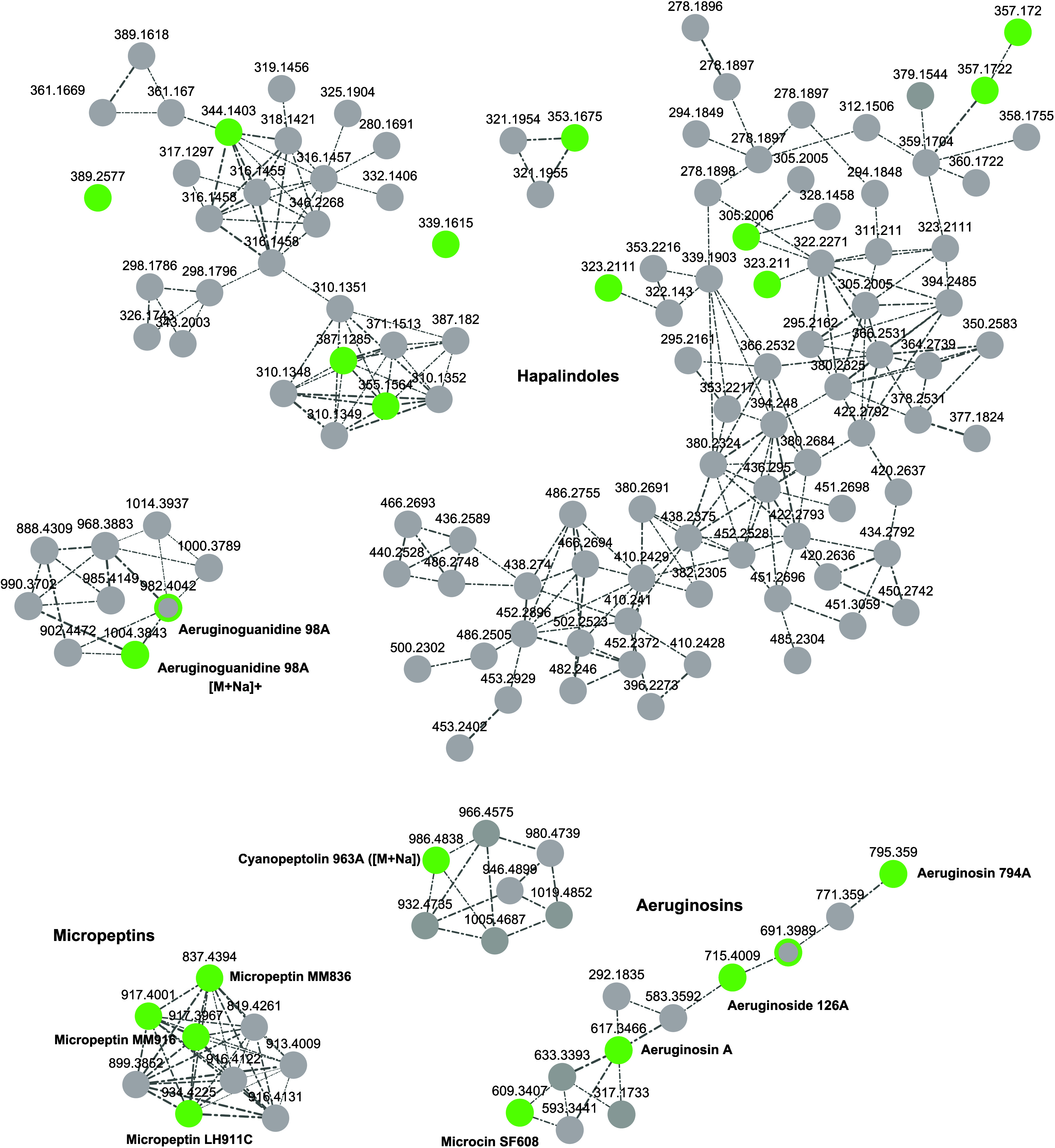
Feature Based Molecular Networking (FBMN) analysis of a sample set of cyanobacterial extracts with major compounds known *a priori* to the authors for chemical space visualization: positive mode data. The compound annotation within this subset of the networking analysis was achieved through structure database search using the SIRIUS platform. Nodes are connected for cosine scores ≥0.7, with edge width correlating to cosine scores. Annotations derived from structure database searches are highlighted in green. Features without annotation, either corresponding to cyanobacterial SMs or uncharacterized compounds in general, are shown in gray. Shown clusters represent compound families lacking spectral reference data needed for confident annotation.

**Figure 5:**
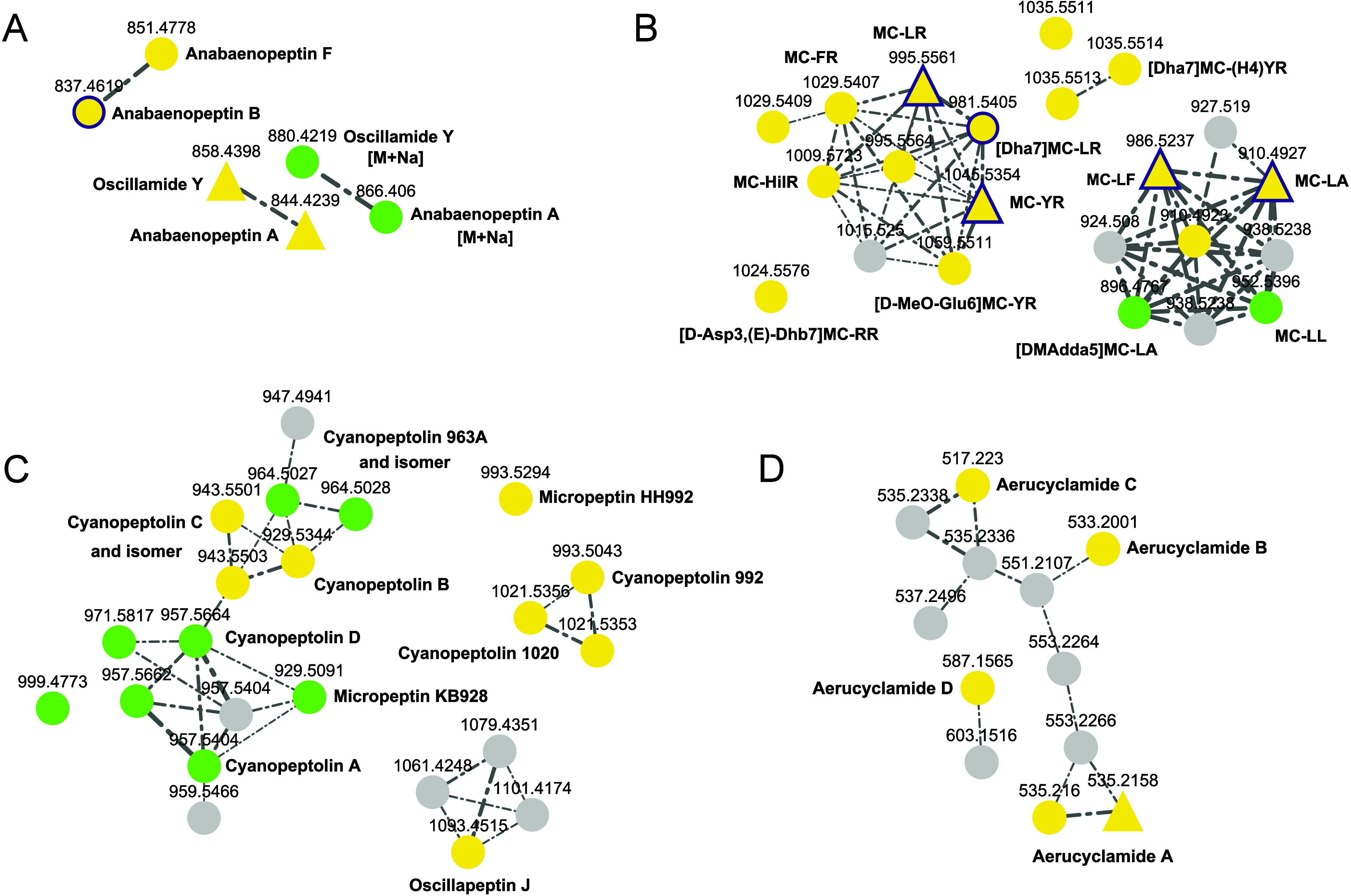
Feature Based Molecular Networking (FBMN) analysis (subset of the networking analysis; overview Figure S1) of an uncharacterized sample set of cyanobacterial extracts (Table S3) for chemical space visualization. Compound annotation was achieved through spectral library matching and structure database search using the SIRIUS platform. Nodes are connected for cosine scores ≥0.6, with edge width correlating to cosine scores. Clusters of the four main compound classes are highlighted. Annotations obtained through spectral library matching are highlighted in yellow, and those derived from structure database searches are shown in green. Triangular nodes indicate spectra previously included in the MassBank database, while nodes outlined in purple represent features additionally annotated via GNPS. Features without annotation, either corresponding to cyanobacterial SMs or uncharacterized compounds in general, are shown in gray. (A) Cluster of anabaenopeptins. (B) Cluster of microcystins, displaying an increased spectral annotation rate compared to previous data sets. (C) Cluster of cyanopeptolins, for which spectral annotations were enabled by HRMS^2^ spectra recorded in this study. (D) Cluster of aerucyclamides, likewise demonstrating enhanced annotation coverage.

### Case Study

As a case study, HPLC-HRMS data obtained from different cyanobacterial strains (Table S3) were used as an unbiased test set to assess which cyanobacterial SMs could be identified through spectral library matching without prior knowledge. Our aim was to evaluate whether the inclusion of new HRMS^2^ reference data could increase the confidence in the identification of known compounds and facilitate the annotation of previously uncharacterized chemical space of a data set (Figure S1, Figure 5A–D). In total, FBMN analysis revealed 351 nodes, of which 300 were organized into clusters. Of these, 34 nodes were annotated via spectral library matching, while 16 were annotated through structure database searches. Notably, 25 of these 34 annotations could only be achieved when using the reference spectra generated in this study (Figure 5, Table S6). Two compounds were annotated with the newly acquired reference spectra, as well as user-contributed spectra available on the GNPS platform. The inclusion of the new reference spectra led to a substantial increase in the overall annotation rate from roughly 2% to 10%. In conclusion, our newly acquired reference spectra were indispensable for correct annotation, as no other suitable data were available to support the identification of these compounds.

In both the proof-of-concept study and the case study, several structure database annotations were associated with features located in clusters already containing high-confidence spectral library matches, further supporting the reliability of these annotations. When cluster-based annotation relationships are considered, like in the cyanopeptolin cluster (Figure 5C), the total annotation coverage across the data set increased to approximately 13%. Moreover, clusters corresponding to compound classes such as anabaenopeptins, microcystins, and aerucyclamides could have already been identified based on existing reference data in public databases (Figure 5A–C). In contrast, the cyanopeptolin cluster and planktocyclin were reliably annotated only after incorporating the newly generated reference spectra. These high-confidence annotations can serve as anchors for the interpretation of additional nodes, allowing further compounds to be recognized based on exact mass matches or lower-confidence *in silico* predictions. This approach highlights how reliable reference spectra not only improve the confidence of individual annotations but also enable the systematic exploration of a previously uncharacterized chemical space. High-confidence annotations derived from spectral library matching serve as key nodes within the molecular networks. These key nodes increase the reliability of annotating connected features via a structure database search (e.g., SIRIUS platform), or even when such features are supported only by exact mass matches (suspect lists), which represent a lower-confidence level of compound annotation, thereby enhancing dereplication.^37,38^

A spectral-library similarity >80% was defined as a high-quality MS^2^ match. When this threshold was combined with the FBMN analysis—i.e., when the annotated compound was found within a cluster of structurally related SMs—a similarity >70% was also considered to provide good-confidence annotation. Scores ranging from 50% to 70% were regarded as “tentatively identified”, especially when the accurate mass measurement corroborated the proposed annotation. Features with a similarity <50% were excluded from further discussion, even though, as discussed above, the microginins exemplify a case where low thresholds might be linked to suboptimal acquisition parameters. This latter example, in particular, highlights that manual inspection and interpretation of the HRMS^2^ data remain necessary; a careful, manual annotation of MS^2^ spectra can provide stronger evidence for a confident identification.

## Conclusions and Implications

The workflow to systematically record and process highly curated HRMS^2^ reference spectra for deposition in MassBank EU allowed us to increase the number of available spectra for SMs from cyanobacteria from 11 to 150. This first attempt to generate reference spectra for metabolites in semipurified extracts rather than only from reference materials required adjustments to the open-source scripts and a significant time commitment for manual quality control steps.

The new reference spectra are publicly available and open access through MassBank EU and are automatically linked to other major spectral libraries, including the Human Metabolome Database (HMDB), MetaboLights, MassBank of North America (MoNA), PubChem, the US EPA CompTox Dashboard, NORMAN Database System, and RforMassSpectrometry. They are also used as a data source for searches in tools like GNPS and are included in the Metabolomics Spectrum Identifier Resolver. The reference spectra serve the purpose to obtain spectral matches in high-throughput non-target screening to identify tentative candidates and to improve dereplication efficiency. Compounds cannot be identified based on MS fragmentation alone, but the shorter list of matched tentative candidates can then be further investigated, e.g., by getting other reference samples from different sources to verify the spectra and retention times. Moreover, tentative candidates can serve as key nodes in molecular networking approaches available through the GNPS platform, such as Classical Molecular Networking or FBMN workflows. This facilitates the systematic exploration of a previously uncharacterized chemical space and supports accelerated dereplication strategies. The proof-of-concept and the case study revealed two key aspects: (i) several compounds were annotated through structure database searches but lacked corresponding experimental reference spectra, underscoring the need to further expand spectral databases; and (ii) in some cases, no annotation based on spectral library matching was obtained despite both the compound being present in the sample and a reference spectrum being available, highlighting that reference spectra may not always be perfectly representative or may not account for adduct formation influencing fragmentation behavior. Together, these findings emphasize the importance of continuously extending and refining the reference data set in future work.

## Experimental Section

### General Experimental Procedures

LC–MS/MS data for the different experiments were acquired as described in the subsections below. The software used for data preparation and processing is also listed and described in detail in the corresponding sections. No additional general experimental procedures were employed.

### Sample Preparation

The samples were provided either as reference materials or biomass extracts containing known SMs from cyanobacteria based on previous NMR or MS analyses by collaborators of CyanoMetDB (see acknowledgments). The samples were provided in aqueous phase or containing up to 100% methanol, whereas the latter were diluted with nanopure water to match chromatographic conditions. The teams provided metadata for each sample including the cyanobacterial species as the producing organism, structural information on the metabolites (CyanoMetDB_ID, molecular formula, SMILES), and the expected precursor ion (adduct, charge state, *m*/*z*). Materials were stored at −20 °C until analysis.

### HPLC-HRMS/MS Analysis

For 336 compounds, HRMS data acquisition was performed on an Orbitrap Exploris 240 mass spectrometer equipped with a heated ESI interface coupled to a Dionex UltiMate 3000 RS pump HPLC system (Thermo Fischer Scientific). The following chromatographic parameters were used: XBridge C18 column (50 × 2.1 mm, 3.5 μm, 100 Å, Phenomenex), binary gradient with solvent A being water and solvent B being methanol (0.1% formic acid in A and B) at 0.2 mL/min from 10% to 50% B in 4 min, from 50% to 95% B for 13 min, and 95% B for 8 min. HRMS data acquisition: positive (pos.) and negative (neg.) ionization modes, ESI spray voltages of 3.5 kV and −2.5 kV, capillary temperature of 320 °C, tube lens of 70 V, sheath gas flow rate of 40 L/min, and auxiliary gas flow rate of 10 L/min. Full scan spectra were acquired from *m*/*z* 100 to 1000 with a resolution of 120,000 at *m*/*z* 200. MS^2^ spectra were acquired in data-dependent acquisition mode (dd-MS^2^), a range of 9 MS^2^ experiments in each pos. and neg. mode with HCD energies of 15%, 20%, 25%, 30%, 40%, 50%, 60%, 70%, and 80% at a resolution of 15,000 at compound-specific varying *m*/*z* (100–1000). The isolation window was 1.0 Da, and the automatic gain control (AGC) target was 5 × 10^4^. All measurements were recorded in profile mode with two consecutive scan range settings: first with an automatic scan range, starting at the precuror *m*/*z* and scanning down to 1/15 of the precursor *m*/*z*, and second fixing the starting *m*/*z* to 40, without considering the precursor *m*/*z*. In total, for each compound, 36 unique spectra types were considered with 9 collision energies, 2 ionization modes (pos. and neg.), and 2 MS^2^ scan modes (auto and first fix mass *m*/*z* 40) (Figure 6).

In addition, 54 compounds were analyzed using an Acquity UPLC HSS T3 analytical column (100 × 3 mm, 1.8 μm, Waters) to improve the chromatographic separation. The following chromatographic parameters were used: binary gradient with solvent A being water and solvent B being methanol (0.1% formic acid in A and B) at 0.2 mL/min from 15% to 100% B in 25 min, followed by 98% B for 6 min at 0.65 mL/min. Compared to the method described above, normalized collision energies of 15–70% were used with an additional 35% step. Otherwise, identical HPLC and HRMS/MS settings on the Orbitrap Exploris 240 instrument were used as described above. In addition to [M + H]^+^ and [M – H]^−^, [M + 2H]^2+^ precursors were considered increase chances for adequate HRMS^2^ spectra.

Additionally, 75 compounds were analyzed with slightly different settings on an QExactive Plus mass spectrometer equipped with a heated ESI interface coupled to an UltiMate 3000 HPLC system. The following chromatographic parameters were used: Kinetex C18 column (50 × 2.1 mm, 2.6 μm, 100 Å, Phenomenex), a binary gradient equivalent to the previously described method, with MeCN replacing MeOH, was used at 0.4 mL/min. HRMS data acquisition setting: pos. and neg. ionization modes, ESI spray voltages of 3.5 and −2.5 kV, capillary temperature of 350 °C, sheath gas flow rate of 50 L/min, and auxiliary gas flow rate of 12.5 L/min. Full scan spectra were acquired from *m*/*z* 100–1250 with a resolution of 70,000 at *m*/*z* 200, an AGC target of 1 × 10^6^, and a maximal injection time of 50 ms. MS^2^ spectra were acquired in dd-MS^2^ mode with an HCD range the same as that for the other measurements from 15% to 80% at a resolution of 17,500 at *m*/*z* 200, an AGC of 2 × 10^5^, and a maximal injection time of 50 ms.

**Figure 6:**
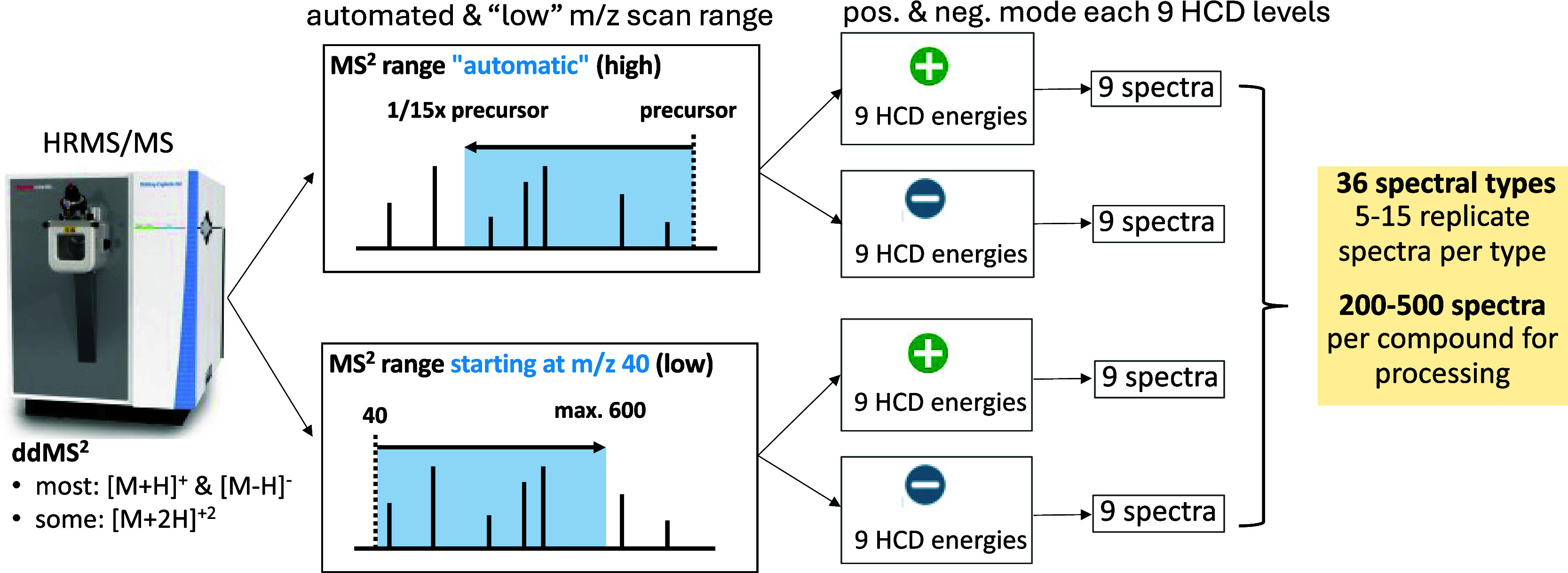
Illustration of MS instrument settings, including 2 scan range modes with automatic settings adjusted for each precursor or fixed first mass at *m*/*z* 40, positive and negative ionization mode with 9 collision energies in each mode (15, 20, 25, 30, 40, 50, 60, 70, 80% NCE) to generate up to 36 unique spectral types with 5–15 relicates per type, and a total of 200–500 spectra per compound for data processing.

### Data Processing

The raw data files were converted to mzML with ProteoWizard MSConvert (Version 3.0) using vendor centroiding.^39^ Metadata of the analyzed materials and compounds was prepared in a CSV table containing the CyanoMetDB compound ID, the compound name, SMILES code, molecular formula, and retention time. A settings file was prepared, defining the processing options and generic metadata to be added to the database spectra. The raw data files and experimental settings file were used as input for a data processing workflow based on RMassBank^25^ with extensions to further improve data quality and simplify manual curation (https://github.com/meowcat/RMassBank-scripts/).

Briefly, the data processing workflow consisted of the following steps: (1) Extraction of MS^2^ spectra based on the compounds specified in the metadata. (2) Assignment of molecular formula to each fragment with large tolerance, using a ±15 ppm window and a ±10 ppm window for low mass range (*m*/*z* ≤ 120) and high mass range. (3) Calculation of recalibration curve of mass error against *m*/*z* values with uniquely assigned fragments, using peaks with intensities ≥ 10^3^. (4) Recalibration of all spectra with calibration curve. (5) Subformula reassignment with smaller tolerance (less than 5 ppm deviation). (6) Unassigned fragments were checked allowing for N_2_ and/or O addition to detect fragment adducts with collision gas. (7) Fragments occurring in at least two spectra were retained. (8) To ensure the accuracy of the extracted spectra, in particular to eliminate spurious formula matches of low-mass fragments, an orthogonal analysis based on the EIC correlation was performed. For all precursor and fragment ions, EICs were extracted, and the correlation/dot product of the fragment to precursor ion EIC was calculated. Results from formula assignment were used to automatically determine a cutoff for EIC correlation: the cutoff was chosen such as to maximize the F_1.5_ score for predicting formula match from EIC correlation. (9) Spectra were reviewed in a graphical user interface (Figure 7), which allowed us to review the formula match and EIC correlation for individual fragment of each spectrum, as well as to include or exclude spectra or entire compounds, or adjust correlation thresholds to a custom value per spectrum or compound if required. A quality threshold was applied based on the EIC correlation of fragments and the precursor. In the spectral plot, all fragments with poor EIC match (below the quality cutoff) were plotted in red in case they were assigned a molecular formula or in black if no molecular formula was assigned. Both types were removed and and thus not part of the spectrum in the final export. Fragments illustrated in green matched with precursor EIC, but have no molecular formula assigned, which can occur when a second precursor is in the isolation window or at the bottom of the mass range outside the well-calibrated domain. These fragments were included, but called for review of the purity of the MS^2^ spectrum. For standard reference materials that could be measured with appropriately high concentrations and purity, a stricter EIC match threshold could be applied. When working with compounds at a large range of intensities from biomass extracts or semipurified extract fractions, individual attention was given to each compound to make a case by case decision on the threshold for eliminating fragments.

**Figure 7:**
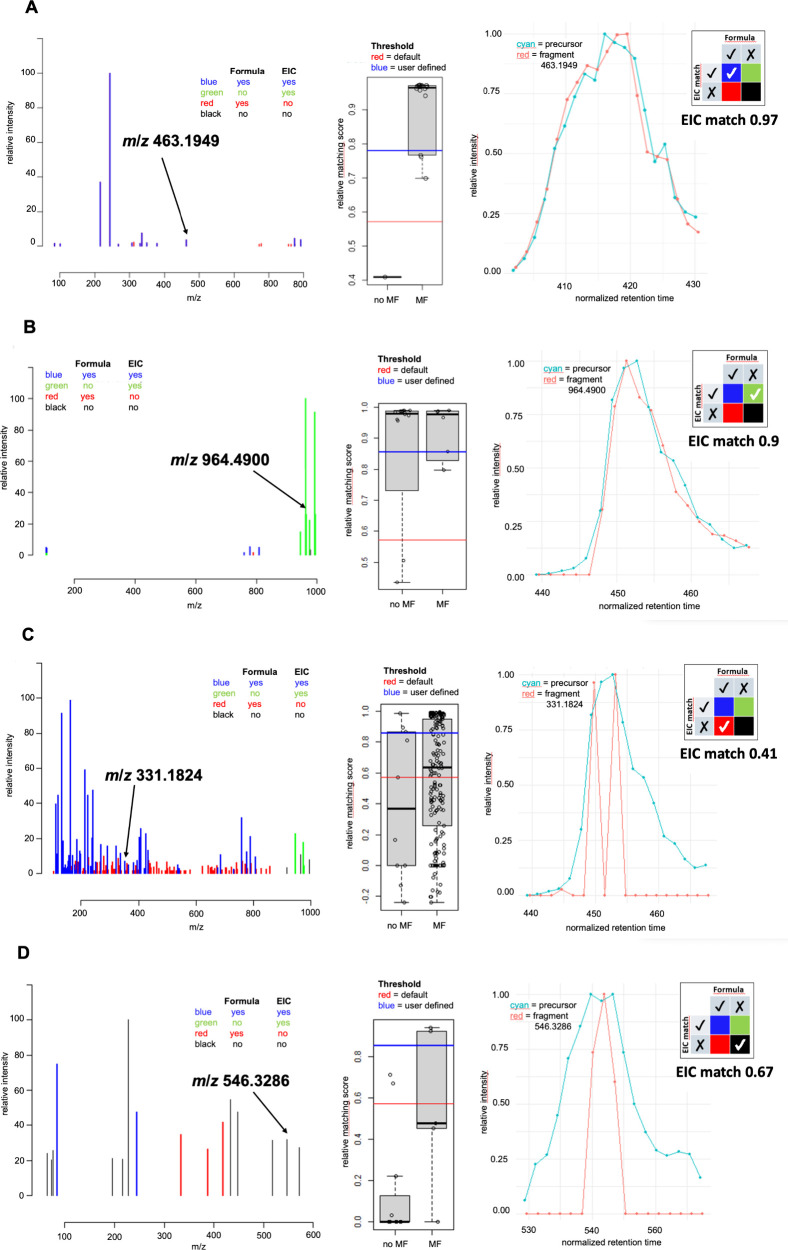
Examples of data represented in the viewer graphical user interface comparing the correlation of fragment to precursor ion EIC. All fragments in the mass spectrum (left) are categorized (A) with molecular formula assigned and with good EIC (blue), (B) with no molecular formula assigned and good EIC match (green), (C) with molecular formula assigned and poor EIC match (red), and (D) with no molecular formula assigned and poor EIC match (black). The range of fit of the relative EIC match for all fragments with and without molecular formula (MF) assigned are shown in a box plot (middle), with the quality cutoff for a good EIC match in red being the viewer default and blue being a user-defined threshold selected for each compound. The precursor EIC (cyan) can be compared with each fragment EIC (red) as relative intensity against retention time (right).

Finally, all selected spectra were exported to MassBank records, combining processed spectra data with experiment metadata from the settings file (e.g., MS type MS,^2^ chromatographic details), and compound metadata retrieved from CyanoMetDB and Internet services (NCI Cactus Chemical Identifier Resolver: https://cactus.nci.nih.gov/; PubChem (PubChem REST API): https://pubchem.ncbi.nlm.nih.gov; EPA CCTE with API-Key set manually: https://www.epa.gov/comptox-tools/computational-toxicology-and-exposure-apis-data-domains; the legacy Chemspider API (formerly available at http://legacy.chemspider.com/) and now under https://developer.rsc.org/; Chemical Translation Service (Fiehn Lab): https://cts.fiehnlab.ucdavis.edu). Each record contains the team (author) and their prior evaluation of the confidence level of the reported compound following recommendations by Schymanski et al.^6^ Level 1 was used when the compound in the supplied material for analysis was at a reference material purity (>95%) and previous MS^2^ annotation and NMR studies confirmed the identity. Level 2a was used when the compound’s spectrum could be matched with an available reference spectrum of an external library, and Level 2b was used when and previous MS^2^ annotation confirmed the identity in the absence of a library match. The identification may have been done by manual annotation of MS^2^ spectra recorded by the collaborating laboratories. When a peer-reviewed reference was available presenting the annotation, a citation was also included in the respective MassBank record. Level 3 was used when the compound identity was inconclusive regarding other structural isomers of the same compound class from previous HRMS^2^ annotations of the same sample material. In these cases, the same MassBank record was associated with all of the isomer names. For all records derived from non-reference materials, the spectra were marked with “TENTATIVE” in the record title, in line with other existing non-reference records in MassBank.

### HPLC-HRMS/MS Data Acquisition for Proof-of-Concept and Case Study

HRMS data analysis was carried out on 15 cyanobacterial biomass extracts selected based on *a priori* knowledge of their SMs, with reference compounds included to evaluate annotation and recovery rate for a proof-of-concept study (**A**) and on 4 additional cyanobacterial biomass extracts selected for a case study (**B**). The data set comprised extracts from (**A**) *Fischerella* sp. (ambigols,^40^ tjipanazoles^41^), *Tolypothrix* sp. (PCC9009: cyanobacterin and analogues),^26^ *Nostoc* sp. (nostotrebin 6 and congeners,^42^ cryptophycins), *Hapalosiphon* sp. (hapalindoles^32^), *Limnothrix* sp. (acutiphycin), *Microcystis* sp. (aerucyclamides, microginins, microcystins^43,44^ ), *Planktothrix* sp. (anabaenopeptins), *Cylindrospermum* sp. (cylindrofridins), and *Scytonema* sp. (Scyptolin A and B) (Table S2) and (**B**) from *Microcystis* sp. (PCC7806: microcystins, cyanopeptolins, cyclamides), *Planktothrix* sp. (K-0576: microcystins, anabaenopeptins), and *Dolichospermum* sp. (NIVA-CYA 269/6: microcystins, cyanopeptolins, cyclamides) (Table S3).^45^ The HRMS data acquisition was performed on either a (**A**) Q Exactive Plus mass spectrometer or (**B**) Orbitrap Exploris 240 mass spectrometer, both equipped with a heated ESI interface coupled to an UltiMate 3000 HPLC system. The following chromatographic parameters were used: Kinetex C18 column (50 × 2.1 mm, 2.6 μm, 100 Å, Phenomenex), (**A**) binary gradient from 5% to 100% MeCN in H_2_O (0.1% formic acid each) at 0.4 mL/min in 16 min, 100% MeCN for 4 min; (**B**) binary gradient 7% for 3 min, from 7% to 30% in 4 min, and from 30% to 100% in 23 min MeOH in H_2_O (0.1% formic acid each) at 0.255 mL/min, 100% MeOH for 7 min. HRMS data acquisition settings: (**A**) pos. and neg. ionization modes, ESI spray voltages of 3.5 and −2.5 kV, capillary temperature of 350 °C, sheath gas flow rate of 50 L/min, and auxiliary gas flow rate of 12.5 L/min; (**B**) pos. ionization mode, ESI spray voltage of 3.5 kV, capillary temperature of 320 °C, sheath gas flow rate of 40 L/min, and auxiliary gas flow rate of 10 L/min. Full scan spectra were acquired from (**A**) *m*/*z* 133.4 to 2000 with a resolution of 35,000 at *m*/*z* 200, automated gain control (AGC) of 5 × 10^5^, and maximal injection time of 120 ms or (**B**) *m*/*z* 110 to 1500 with a resolution of 120,000 at *m*/*z* 200, AGC of 2.5 × 10^5^, and maximal injection time of 50 ms. MS^2^ spectra were acquired (**A**) in data-dependent acquisition mode (dd-MS^2^), stepped energies of 30, 60, and 75 eV (resulting at 55 eV), a resolution of 17500 at *m*/*z* 200, an AGC of 2 × 10^5^, and a maximal injection time of 75 ms and (**B**) in data-dependent acquisition mode (dd-MS^2^) with CyanoMetDB as the targeted mass list, 3 MS^2^ experiments with HCD 15%, 30%, and 45%, a resolution of 15,000 at *m*/*z* 200, an AGC set to standard (set in automated fashion dependent on scan type), and a maximal injection time set to auto (system sets the maximum injection time available according to the transition length, optimizing between sensitivity and scan speed). A TopN experiment (*N* = 5, loop count 5) was implemented for triggering the dd-MS^2^ acquisition for both (**A** and **B**).

### Data Processing for Proof-of-Concept Data

#### File Conversion

Raw mass spectrometry data files were converted from.RAW to .mzML format using MSConvert from ProteoWizard (version 3.0).^39^ A scan polarity filter was used during data conversion to separate positive ion mode scans from negative ion mode scans, facilitating a more targeted analysis in subsequent data analysis steps.

#### Feature-Based Molecular Networking (FBMN) and SIRIUS Analysis

To facilitate FBMN analysis, the converted MS data were processed using MZmine (4.2) with a workflow designed and executed through the MZWizard tool to automate the feature extraction and alignment.^46^ For mass detection, the following parameters were used to discard low-intensity signals: noise level thresholds of 6.00 (MS) and 0.00 (MS^2^) . Chromatogram building was performed with the following parameters: 4 minimum consecutive scans, minimum absolute height of 1.0 × 10^6^, and *m*/*z* tolerance of 10 ppm.^47^ Chromatographic peaks were smoothed using a Savitzky–Golay filter (window size 5 points) to reduce noise and refine the peak shape. The Join Aligner module for peak alignment was used with the following parameters: retention time tolerance of 0.4 min and *m*/*z* tolerance of 5 ppm. MGF files were exported from MZmine 4.2 for analysis in SIRIUS. Molecular formula determination, molecular structure database searches, and spectral library matching were conducted using SIRIUS 6.0.1 and 6.3.2 (CSI:FingerID).^14–16^ For the structure database search, a custom suspect database was compiled from the SMILES strings provided by the current version of CyanoMetDB (version 3.0).^10,48^ Spectral library matching was restricted to the following two custom libraries of reference spectra: (i) spectra from the MassBank database (accessed March 2025) and (ii) spectra acquired and recorded within this study.

The following settings were applied in the compute dialogue. Global configurations included: Orbitrap as instrument with an HRMS^2^ mass accuracy of 5 ppm; Fallback Adducts set to [M + H]^+^, [M + Na]^+^, [M + K]^+^, and [M – H_2_O + H]^+^ for pos. mode and [M – H]^−^ and [M + CH_2_O_2_ – H]^−^ for neg. mode; Search DBs included the custom/suspect databases and the default SIRIUS databases PubChem, CHEBI, COCONUT, GNPS, HSDB, LOTUS, Maconda, SuperNatural, and TeroMOL. Spectral library searching was performed using default settings. Molecular formula identification was conducted using the *de novo* + bottom-up strategy, with the element filter set to default values and Br and Cl additionally allowed and autodetection of Mg, B, Fe, Zn, and Se disabled. The analysis results were exported, and summaries of the structure database searches and spectral library matching were incorporated into the FBMN analysis during visualization in Cytoscape. All annotations were manually reviewed prior to network visualization to ensure their plausibility and consistency. The molecular networks for FBMN analysis were generated using GNPS (http://gnps.ucsd.edu).^13^ All HRMS^2^ fragment ions within a 17 Da window of the precursor *m*/*z* were excluded from the data set. HRMS^2^ spectra were further filtered to select the six most prominent fragment ions within a 50 Da window. Precursor ion mass tolerance was set to 0.02 Da, and the same tolerance was applied to HRMS^2^ fragment ions. Networks were constructed by retaining edges with a cosine score >0.6 and a minimum of four matched peaks. Edges were retained only if both nodes appeared in each other’s top ten most similar nodes list. The maximum size of any molecular family was limited to 100, and the lowest scoring edges were removed until each molecular family was below this threshold.^13,49^ The resulting molecular networks were visualized and analyzed using Cytoscape (version 3.10.2).^50,51^

## Supplementary Material





## Data Availability

The spectral data can be found in MassBank EU (https://massbank.eu/MassBank/) and the repository 10.5281/zenodo.3378723 (release 03/2026), and accession numbers can be found in extended Table S4.

## Notes

List of MassBank EU reference spectra of cyanobacterial specialized metabolites before 2025; strains for the proof-of-concept study and case study; list of 150 specialized metabolites added to MassBank EU herein; library matching annotation results for proof-of-concept and case studies (PDF)
Additional metadata–extended Table S4 (XLSX)
