# Supplementary material for: Curation of Mass Spectrometry Reference Data for Improved Identification and Dereplication of Cyanobacterial Specialized Metabolites
Source: J Nat Prod. 2026 Apr 23;89(5):1499–512. doi: 10.1021/acs.jnatprod.6c00107 (PMC13200231; doi:10.1021/acs.jnatprod.6c00107)
Supplement: Supplementary file 1 [file np6c00107_si_001.pdf]

Curation of mass spectrometry reference data for improved identification and dereplication of cyanobacterial specialized metabolites

Franziska Schanbacher[1,‡], Anne Dax[2,‡], Michele A. Stravs[2], Timo H. J. Niedermeyer[1], Elisabeth M.-L. Janssen[2*]

[1] *Freie Universität Berlin, 14195 Berlin, Germany*

[2]*Eawag, Swiss Federal Institute of Aquatic Science and Technology, 8600 Dübendorf, Switzerland*

‡co-first authorship

* *Corresponding author* Email: Elisabeth.Janssen@eawag.ch

SUPPORTING INFORMATION

The electronic supporting information contains 13 pages including 6 tables.

Table S1. Cyanobacterial SMs in MassBank EU deposited before 2025 (last deposition 2020) with 14 unique compounds and 215 unique $HRMS^2$ spectra.

compound name	MassBank EU assession numbers	precursor ion	resolution	HCDs (%)	deposition date	No spectra
**Microcystin-LR (MCLR)**	MSBNK-Eawag-EA299201 to MSBNK-Eawag-EA299217	M+H	7500	35, 15, 30, 45, 60, 75, 90	14.01.2014	7
29 spectra	MSBNK-Eawag-EA299208 to MSBNK-Eawag-EA299214	M+H	15000	15, 30, 45, 60,75, 90, 35	14.01.2014	7
	MSBNK-Eawag-EQ299201 to MSBNK-Eawag-EQ299209	M+H	17500	15, 30,45, 60,75,90, 120, 150, 180	03.02.2020	9
	MSBNK-Eawag-EQ299251 to MSBNK-Eawag-EQ299256	M-H	17500	15, 30, 45, 60, 75, 90	03.02.2020	6
**Microcystin-LA (MCLA)**	MSBNK-Eawag-EQ324601 to MSBNK-Eawag-EQ324609	M+H	17500	15, 30,45, 60,75,90, 120, 150, 180	03.02.2020	9
15 spectra	MSBNK-Eawag-EQ324651 to MSBNK-Eawag-EQ324656	M-H	17500	15, 30, 45, 60, 75, 90	03.02.2020	6
**Microcystin-LF (MCLF)**	MSBNK-Eawag-EQ324701 to MSBNK-Eawag-EQ324709	M+H	17500	15, 30,45, 60,75,90, 120, 150, 180	03.02.2020	9
18 spectra	MSBNK-Eawag-EQ324751 to MSBNK-Eawag-EQ324759	M-H	17500	15, 30,45, 60,75,90, 120, 150, 180	03.02.2020	9
**Microcystin-LY (MCLY)**	MSBNK-Eawag-EQ324801 to MSBNK-Eawag-EQ324809	M+H	17500	15, 30,45, 60,75,90, 120, 150, 180	03.02.2020	9

18 spectra	MSBNK-Eawag-EQ324851 to MSBNK-Eawag-EQ324859	M-H	17500	15, 30,45, 60,75,90, 120, 150, 180	03.02.2020	9
**Microcystin-LW (MCLW)**	MSBNK-Eawag-EQ324901 to MSBNK-Eawag-EQ324909	M+H	17500	15, 30,45, 60,75,90, 120, 150, 180	03.02.2020	9
18 spectra	MSBNK-Eawag-EQ324951 to MSBNK-Eawag-EQ324959	M-H	17500	15, 30,45, 60,75,90, 120, 150, 180	03.02.2020	9
**Microcystin-RR (MCRR)**	MSBNK-Eawag-EQ325001 to MSBNK-Eawag-EQ325006	M+H	35000	15, 30, 45, 60, 75, 90	25.08.2015	6
12 specrta	MSBNK-Eawag-EQ325051 to MSBNK-Eawag-EQ325054	M-H	17500	15, 30, 45, 60	03.02.2020	4
	MSBNK-Eawag-EQ325056	M-H	35000	90	25.08.2015	1
**Microcystin-YR (MCYR)**	MSBNK-Eawag-EQ325101 to MSBNK-Eawag-EQ325109	M+H	17500	15, 30,45, 60,75,90, 120, 150, 180	03.02.2020	9
18 spectra	MSBNK-Eawag-EQ325151 to MSBNK-Eawag-EQ325159	M-H	17500	15, 30,45, 60,75,90, 120, 150, 180	03.02.2020	9
**[D-Asp3,E-Dhb7]-Microcystin-RR**	MSBNK-Eawag-EQ435801 to MSBNK-Eawag-EQ435809	M+H	17500	15, 30,45, 60,75,90, 120, 150, 180	03.02.2020	9
14 spectra	MSBNK-Eawag-EQ435851 to MSBNK-Eawag-EQ435855	M-H	17500	15, 30,45, 60,75	03.02.2020	5
**Nodularin (Nodularin-R)**	MSBNK-Eawag-EQ325203 to MSBNK-Eawag-EQ325206	M+H	35000	45, 60, 75, 90	25.08.2015	4
10 spectra	MSBNK-Eawag-EQ325251 to MSBNK-Eawag-EQ325256	M+H	35000	15, 30, 45, 60, 75, 90	25.08.2015	6

**Anabaenopeptin A**	MSBNK-Eawag-EQ435601 to MSBNK-Eawag-EQ435609	M+H	17500	15, 30, 45, 60, 75, 90, 120, 150, 180	03.02.2020	9
15 spectra	MSBNK-Eawag-EQ435651 to MSBNK-Eawag-EQ435656	M-H	17500	15, 30, 45, 60, 75, 90	03.02.2020	6
**Anabaenopeptin NZ857**	MSBNK-Eawag-EQ435901 to MSBNK-Eawag-EQ435909	M+H	17500	15, 30, 45, 60, 75, 90, 120, 150, 180	03.02.2020	9
15 spectra	MSBNK-Eawag-EQ435951 to MSBNK-Eawag-EQ435956	M-H	17500	15, 30, 45, 60, 75, 90	03.02.2020	6
**Anabaenopeptin B**	MSBNK-Eawag-EQ436101 to MSBNK-Eawag-EQ436109	M+H	17500	15, 30, 45, 60, 75, 90, 120, 150, 180	03.02.2020	9
15 spectra	MSBNK-Eawag-EQ436151 to MSBNK-Eawag-EQ436156	M-H	17500	15, 30, 45, 60, 75, 90	03.02.2020	6
**Oscillamide Y**	MSBNK-Eawag-EQ436001 to MSBNK-Eawag-EQ436009	M+H	17500	15, 30, 45, 60, 75, 90, 120, 150, 180	03.02.2020	9
9 spectra						
**Aerucyclamide A**	MSBNK-Eawag-EQ436301 to MSBNK-Eawag-EQ436309	M+H	17500	15, 30, 45, 60, 75, 90, 120, 150, 180	03.02.2020	9
9 spectra						

Table S2. Strains used in the proof-of-concept study and their biomass extracts (post-freeze drying, 50:50 by volume methanol:water and 80:20 by volume methanol:water at a solvent-to-biomass ratio of 20 mL/g, sonication-assisted, supernatants combined, *extract dried in vacuo,* reconstitution in 80:20 by volume methanol:water). The major compounds were known a priori to the authors. Reference compounds were included to allow assessment of annotation and recovery rates in subsequent analyses within the proof-of-concept study.

Strain-id	Genus	Expected SMs
id_1	*Fischerella* sp.	ambigols, tjipanazoles
id_2	*Tolypothrix* sp. PCC9009	cyanobacterins
id_3	*Nostoc* sp.	nostotrebins
id_4	*Nostoc* sp.	cryptophycins
id_5	*Hapalosiphon* sp.	hapalindoles
id_6	*Limnothrix* sp.	acutiphycin
id_7	*Microcystis* sp.	aerucyclamides
id_8	*Planktothrix* sp.	anabaenopeptins
id_9	*Planktothrix* sp.	anabaenopeptins
id_10	*Microcystis* sp.	microginins and microcystins
id_11	*Cylindrospermum* sp.	cylindrofridins
id_12	*Scytonema* sp.	scyptolins
id_13	*Microcystis* sp.	microcystins
id_14	*Nostoc* sp.	cryptophycins
id_15	*Microcystis* sp.	microcystins

Table S3. Strains used in the case study and their biomass extracts (post-freeze drying, 70:30 by volume methanol:water, sonication-assisted, dilution with water to 5% methanol), with major compounds postulated by the authors based on manual $HRMS^2$ annotation.

Strain-id	Genus (strain)	postulated SMs
id_16	*Microcystis* sp. PCC7806	microcystins, cyanopeptolins, cyclamides
id_17	*Planktothrix* sp. *K-0576*	microcystins, anabaenopeptin
id_18	*Dolichospermum* sp. *NIVA-CYA 269/6*	microcystins, anabaenopeptin
id_19	*Microcystis* sp. UV006	microcystins, cyanopeptolins, cyclamides

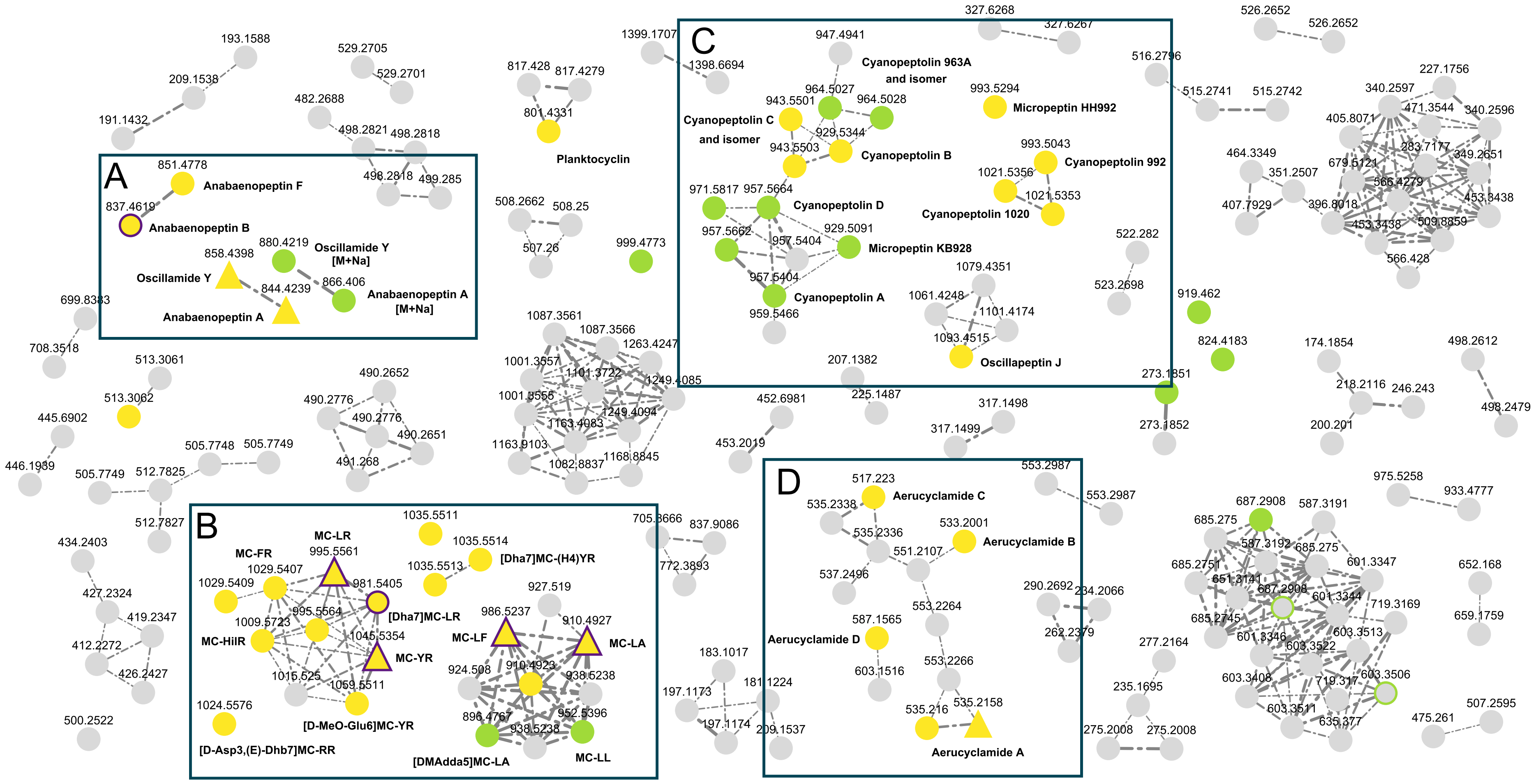

Figure S1. A Feature Based Molecular Networking (FBMN) analysis (overview, subsets **A-D** can be found in the main manuscript) of an uncharacterized sample set of cyanobacterial extracts (Table S3) for chemical space visualization. Compound annotation was achieved through spectral library matching and structure database search using the SIRIUS platform. Nodes connected for cosine scores ≥0.6, edge width correlating to cosine scores. Clusters of the four main compound classes are highlighted. Annotations obtained through spectral library matching are highlighted in yellow, and those derived from structure database searches in green. Triangular nodes indicate spectra previously included in the

MassBank database, while nodes outlined in purple represent features additionally annotated via GNPS. Features without annotation, either corresponding to cyanobacterial SMs or uncharacterized compounds in general, are shown in gray.

Table S4. Cyanobacterial SMs represented with 150 unique compounds and 2911 unique HRMS[2] spectra (at 17500 resolution) in MassBank EU added herein and 11 compounds previously in Massbank (before 2025) represented with new spectra are marked with a hashtag (#), showing the number of spectra from M+2H, M+H and M-H precursor ions with scan mode "auto" and "40" (starting at *m/z* 40), as well as the total number of spectra deposited and the level of confidence for the compound identification.

CyanoMetDB ID	Compound	Molecular Formula	Nr of spectra M+2H	Nr of M+H spectra; auto	Nr of M+H spectra; 40	Nr of M-H spectra; auto	Nr of M-H spectra; 40	Nr of total spectra	Level of confidence
1823 #	MC-LR	C49H74N10O12	9	9	9	9	9	45	1
1802 #	MC-RR	C49H75N13O12	0	9	0	9	0	18	1
1844 #	MC-YR	C52H72N10O13	0	6	0	0	0	6	1
1894 #	MC-LA	C46H67N7O12	0	9	9	9	9	36	1
1916 #	MC-LY	C52H71N7O13	0	9	9	8	8	34	1
1962 #	[D-Asp3,(E)-Dhb7]MC-RR	C48H73N13O12	0	0	0	5	n.a	5	1
1953	[D-Asp3]MC-LR	C48H72N10O12	9	9	0	9	0	27	1
1861	[Dha7]MC-LR	C48H72N10O12	0	9	0	9	0	18	1
1879	MC-HilR	C50H76N10O12	0	9	0	0	0	9	1
1972	[D-Leu1]MC-LY	C55H77N7O13	0	9	0	9	0	18	1
2066	MC-RY	C52H72N10O13	0	9	0	7	0	16	1
861 #	Nodularin-R	C41H60N8O10	0	9	9	9	9	36	1
867 #	Anabaenopeptin B	C41H60N10O9	9	9	0	9	0	27	1
1466 #	Aerucyclamide A	C24H34N6O4S2	0	7	0	0	n.a	7	1
1472	Aerucyclamide B	C24H32N6O4S2	0	9	0	0	n.a	9	1
258	Tychonamide A	C73H107N13O20	0	0	0	9	n.a	9	1
259	Tychonamide B	C72H105N13O19	0	6	0	9	n.a	15	1
653	Brunsvicamide B	C46H66N8O8	0	9	0	4	n.a	13	1
702	Brunsvicamide C	C45H64N8O10	0	9	0	9	n.a	18	1
551	Cyanopeptolin CP990	C49H70N10O12	0	9	9	8	4	30	1
616	Cyanopeptolin CP962	C47H66N10O12	0	9	9	9	9	36	1
642	Cyanopeptolin B	C46H72N8O12	0	18	9	4	0	31	1
983	Carbamidocyclophane A	C38H54Cl4N2O8	0	0	0	4	n.a	4	1
978	Carbamidocyclophane B	C38H55Cl3N2O8	0	0	0	4	n.a	4	1

972	Carbamidocyclophane C	C38H56Cl2N2O8	0	0	0	4	n.a	4	1
969	Carbamidocyclophane D	C38H57ClN2O8	0	0	0	5	n.a	5	1
963	Carbamidocyclophane E	C38H58N2O8	0	0	0	6	n.a	6	1
214	Molassamide	C48H66N8O13	0	7	0	7	n.a	14	1
2277	Molassamide B	C48H65BrN8O13	0	8	0	6	n.a	14	1
2273	Rivulariapeptolide 1185	C61H87N9O15	0	9	0	9	n.a	18	1
2275	Rivulariapeptolide 1121	C56H83N9O15	0	9	0	9	n.a	18	1
2276	Rivulariapeptolide 988	C50H68N8O13	0	9	0	3	n.a	12	1
2364	Cylindrocyclophane B	C38H58O7	0	0	0	5	n.a	5	1
2365	Cylindrocyclophane D	C40H60O8	0	0	0	6	n.a	6	1
2340	Insulapeptolide D	C48H75N9O12	0	9	0	5	n.a	14	1
2341	Insulapeptolide E	C51H72N8O14	0	9	0	9	n.a	18	1
2342	Insulapeptolide F	C50H70N8O14	0	9	0	8	n.a	17	1
2343	Insulapeptolide G	C50H70N8O13	0	9	0	7	n.a	16	1
2344	Insulapeptolide H	C51H72N8O13	0	9	0	7	n.a	16	1
21	Tutuilamide A	C51H69ClN8O12	0	9	0	8	n.a	17	1
155	Balticidin C	C75H121N11O36	0	9	0	0	n.a	9	1
1213	Gallinamide A	C31H52N4O7	0	9	0	0	n.a	9	1
1240	Aeruginosin NOL3	C30H48N6O6	0	9	9	3	4	25	1
1339	Acutiphycin	C27H44O7	0	0	0	8	n.a	8	1
525	Nostotrebin 6	C50H38O10	0	7	0	0	n.a	7	1
923	Nostocyclopeptide A2	C40H54N8O9	0	9	9	9	4	31	1
924	Kasumigamide	C40H50N8O9	0	8	0	6	n.a	14	1
2367	Nostolactone 4	C25H20O6	0	9	0	8	n.a	17	1
2520	Anhydrocyanobacterin	C23H21ClO5	0	9	0	0	n.a	9	1
760 #	Anabaenopeptin A	C44H57N7O10	0	8	0	7	n.a	15	2a
713 #	Oscillamide Y	C45H59N7O10	0	5	0	4	n.a	9	2a
859	Anabaenopeptin C	C41H60N8O9	0	8	0	7	n.a	15	2a
1118	Bartoloside E	C34H58Cl2O6	0	9	9	7	0	25	2a
993	Bartoloside F	C37H64Cl2O6	0	9	9	7	4	29	2a
1027	Bartoloside G	C36H63ClO6	0	9	9	7	0	25	2a
1031	Bartoloside I	C36H61Cl3O6	0	9	9	7	0	25	2a
1117	Bartoloside J	C34H59ClO6	0	9	9	7	0	25	2a

1079	Bartoloside K	C35H60Cl2O6	0	9	9	0	0	18	2a
352	Lyngbyazothrin A	C62H96N12O19	0	9	0	6	n.a	15	2a
355	Lyngbyazothrin B	C61H94N12O18	0	9	0	6	n.a	15	2a
326	Lyngbyazothrin D	C73H107N13O21	0	9	0	2	n.a	11	2a
248	Tjipanazol D	C18H10Cl2N2	0	0	0	9	n.a	9	2a
255	Tjipanazol I	C18H11ClN2	0	0	0	9	n.a	9	2a
2518	Tjipanazol L	C20H9Cl2N3O2	0	0	0	9	n.a	9	2a
2519	Tjipanazol M	C20H10ClN3O2	0	0	0	8	n.a	8	2a
41	Portoamide A	C74H109N13O22	0	9	9	0	0	18	2a
42	Portoamide B	C73H107N13O21	0	9	9	0	0	18	2a
173	Portoamide C	C62H96N12O19	0	8	9	9	9	35	2a
174	Portoamide D	C61H94N12O18	0	8	9	9	9	35	2a
684	Scyptolin A	C45H69ClN8O14	0	9	0	3	n.a	12	2a
464	Scyptolin B	C52H80ClN9O16	0	7	0	3	n.a	10	2a
190	Scytocyclamide B	C63H110N14O19	0	9	0	6	n.a	15	2a
191	Scytocyclamide C	C63H110N14O18	0	9	0	2	n.a	11	2a
1104	Cryptophycin A	C35H43ClN2O8	0	9	0	6	n.a	15	2a
1469	Aerucyclamide C	C24H32N6O5S	0	18	9	0	0	27	2a
149	Sphaerocyclamide	C46H63N9O11	0	9	9	9	7	34	2a
15	Nocuolin A	C16H30N2O3	0	9	9	9	9	36	2a
900	Cylindrofridin C	C40H61ClO8	0	0	0	5	n.a	5	2a
904	Cyanostatin B	C40H59N5O9	0	7	0	7	n.a	14	2a
961	Cylindrofridin B	C38H59ClO7	0	0	0	5	n.a	5	2a
1282	Namalide B	C29H45N5O7	0	9	9	9	9	36	2a
811	Schizopeptin 791	C42H61N7O8	0	9	9	6	4	28	2a
1481	Hierridin B	C23H40O3	0	9	8	9	9	35	2a
1694	7-Deoxy-Cylindrospermopsin	C15H21N5O6S	0	9	0	9	0	18	2a
2515	Monomethylaetokthonostatin	C42H73N5O7	0	9	0	0	n.a	9	2a
140	Pseudospumigin A	C31H44N6O7	0	9	9	0	0	18	2a
1800	MC-FR	C52H72N10O12	0	7	0	6	0	13	2b
1835	MC-FL	C52H71N7O12	0	9	9	7	8	33	2b
1869	MC-YL	C52H71N7O13	0	9	0	7	0	16	2b
1877	[D-MeO-Glu6]MC-LR	C50H76N10O12	0	9	9	8	7	33	2b

1895	[D-Asp3]MC-RY	C51H70N10O13	0	9	9	9	9	36	2b
1950	[D-Asp3,Dha7]MC-LR	C47H70N10O12	0	9	0	5	n.a	14	2b
1997	[D-Leu1]MC-LR	C52H80N10O12	0	9	9	8	6	32	2b
2036	[D-Met(O)1]MC-LR	C51H78N10O13S	0	5	0	0	0	5	2b
668	Anabaenopeptin 871	C46H61N7O10	0	5	0	6	0	11	2b
805	Anabaenopeptin F	C42H62N10O9	0	9	9	9	9	36	2b
809	Anabaenopeptin 807	C42H61N7O9	0	0	0	4	0	4	2b
467	Cyanopeptolin CP1048	C52H76N10O13	0	9	9	9	9	36	2b
512	Cyanopeptolin 1020	C50H72N10O13	0	9	9	9	9	36	2b
532	Cyanopeptolin 1014	C49H78N10O13	0	9	9	4	4	26	2b
555	Cyanopeptolin 963A	C49H69N7O13	0	0	0	9	0	9	2b
603	Cyanopeptolin C	C47H74N8O12	0	15	0	13	0	28	2b
605	Micropeptin K139	C47H74N10O13	0	9	9	9	9	36	2b
613	Oscillapeptin J	C47H68N10O18S	0	9	9	5	9	32	2b
638	Cyanopeptolin 972	C46H72N10O13	0	9	9	9	9	36	2b
699	Nodulapeptin 879	C45H65N7O11	0	5	0	3	0	8	2b
733	[Met6] Nodulapeptin C	C44H65N7O9S2	0	9	9	6	5	29	2b
735	Nodulapeptin 883a	C44H65N7O8S2	0	4	0	4	0	8	2b
748	Nodulapeptin B	C44H63N7O12S	0	9	0	6	0	15	2b
750	Nodulapeptin C	C44H63N7O11S	0	9	9	9	8	35	2b
752	Nodulapeptin 881a	C44H63N7O10S	0	9	7	6	4	26	2b
2240	Microginin 299A	C45H67ClN6O10	0	9	9	9	9	36	2b
2241	Microginin 299B	C45H66Cl2N6O10	0	9	9	5	5	28	2b
2242	Microginin 299C	C45H68N6O10	0	9	9	9	9	36	2b
975	Microginin FR5	C38H55N5O9	0	7	0	0	0	7	2b
1019	Microginin 761B	C37H52ClN5O10	0	9	0	4	0	13	2b
948	Microginin 757	C39H59N5O10	0	9	0	0	0	9	2b
847	Microginin 770	C41H63N5O9	0	9	9	9	9	36	2b
897	Microginin SD755	C40H61N5O9	0	9	0	0	0	9	2b
898	Microginin 756	C40H61N5O9	0	9	9	9	9	36	2b
947	Nostoginin BN741	C39H59N5O9	0	9	9	0	9	27	2b
1221	Spumigin A	C31H44N6O7	0	9	9	3	3	24	2b
1250	Spumigin D	C30H42N6O7	0	9	9	0	0	18	2b

1256	Spumigin F	C30H40N6O7	0	9	9	0	0	18	2b
1231	Spumigin G	C31H42N6O6	0	9	9	0	0	18	2b
1504	Nostosin A	C22H35N5O5	0	9	9	0	0	18	2b
1503	Nostosin B	C22H37N5O5	0	9	9	0	0	18	2b
2163	Nostocyclopeptide Ncp-E1-L	C39H54N8O10	0	9	9	9	9	36	2b
2165	Nostocyclopeptide Ncp-E2-L	C36H56N8O10	0	9	9	9	9	36	2b
1245	Aeruginosin NAL2	C30H46N6O6	0	9	9	6	6	30	2b
1275	Oscillaginin A	C29H47ClO8N4	0	9	0	7	0	16	2b
1332	Muscoride A	C28H40N4O5	0	9	9	0	0	18	2b
1402	Aerucyclamide D	C26H30N6O4S3	0	9	9	3	0	21	2b
137	Namalide D	C29H45N5O6	0	9	9	8	8	34	2b
260	Muscoride B	C31H41N5O6	0	9	9	0	0	18	2b
266	Anabaenolysin A	C28H38N4O8	0	9	2	9	8	28	2b
421	Planktopeptin BL 1125	C54H79N9O17	0	9	0	9	0	18	2b
939	Planktocyclin	C39H60N8O8S	0	9	0	0	0	9	2b
584	Cyanopeptolin 992	C48H68N10O13	0	9	9	4	0	22	3
912	Microginin GH787	C40H58ClN5O9	0	9	9	8	0	26	3
**Isomer Group_C24H34N6O6S**			0	0	0	9	0	9	3
1471	Microcyclamide 7806A	C24H34N6O6S							
1465	Microcyclamide 7806B	C24H34N6O6S							
**Isomer Group_C42H61N7O10S**			0	9	0	6	0	15	3
813	Nodulapeptin 855a	C42H61N7O10S							
814	Nodulapeptin 855b	C42H61N7O10S							
**Isomer Group_C41H61N5O9**			0	9	9	4	0	22	3
857	Microginin 767	C41H61N5O9							
858	Microginin KR767	C41H61N5O9							
**Isomer Group_C31H44N6O7**			0	9	9	0	0	18	3
1222	Dihydrospumigin K	C31H44N6O7							
1223	Dihydrospumigin L	C31H44N6O7							
**Isomer Group_C31H42N6O7**			0	9	9	4	3	25	3
1228	Spumigin E	C31H42N6O7							
1229	Spumigin K	C31H42N6O7							

1230	Spumigin L	C31H42N6O7							
**Isomer Group_C31H42N6O7**			0	9	9	0	0	18	3
1251	Dihydrospumigin M	C30H42N6O7							
1252	Dihydrospumigin N	C30H42N6O7							
**Isomer Group_C51H78N10O12**			0	0	0	5	5	10	3
1834	[Leu1,D-Asp3]MC-LR	C51H78N10O12							
1911	[Leu1,Dha7]MC-LR	C51H78N10O12							
**Isomer Group_C51H78N10O12**			0	7	0	0	n.a	7	3
1947	[Dha7]MC-(H4)YR	C51H74N10O13							
1948	[D-Asp3]MC-(H4)YR	C51H74N10O13							
1949	[DMAdda5]MC-(H4)YR	C51H74N10O13							
**Isomer Group_C53H74N10O13**			0	7	6	8	7	28	3
2014	[D-Glu(OMe)6]MC-YR	C53H74N10O13							
2015	MCHtyR	C53H74N10O13							
2016	[D-Asp3,D-Glu(OMe)6]MC-HtyR	C53H74N10O13							
**Isomer Group_C50H72N8O13**			0	7	0	0	0	7	3
509	Micropeptin HH992	C50H72N8O13							
510	Micropeptin KB992	C50H72N8O13							
511	Loggerpeptin A	C50H72N8O13							

Table S5. Spectral library matching annotation results obtained for the biomass extracts analyzed in the proof-of-concept study (Table S2). Mass deviation from exact mass in parentheses. Annotations exclusively based on newly recorded reference spectra are highlighted (*).

specialized metabolite (expected)	specialized metabolite (annotated)	*m/z* (accurate mass)	*m/z* (exact mass)	mol. formula	spectral library matching
ambigols, tjipanazoles	Tjipanazole D*	323.0145 [M-H]⁻	323.0148 [M-H]⁻	$C_{18}H_{10}C_{12}N_2$ (Δ 0.9 ppm)	78%
	Tjipanazole I*	289.0539 [M-H]⁻	289.0538 [M-H]⁻	$C_{18}H_{11}ClN_2$ (Δ 0.3 ppm)	77%
Cyanobacterin and analogues	Anhyrocyanobactin (isomers 1 + 2)*	413.1149 [M+H]⁺	413.1150 [M+H]⁺	$C_{23}H_{21}ClO_5$ (Δ 0.2 ppm)	81% / 82%
Nostotrebin 6 and related compounds	Nostolacton 4*	417.1329 [M+H]⁺	417.1333 [M+H]⁺	$C_{25}H_{20}O_6$ (Δ 0.2 ppm)	87%
	Nostotrebin 6*	799.2533 [M+H]⁺	799.2538 [M+H]⁺	$C_{50}H_{38}O_{10}$ (Δ 0.6 ppm)	68%
	(3 isomers/analogues)	799.2505 [M+H]⁺	799.2538 [M+H]⁺	$C_{50}H_{38}O_{10}$ (Δ 4.1 ppm)	66%
		799.2531 [M+H]⁺	799.2538 [M+H]⁺	$C_{50}H_{38}O_{10}$ (Δ 0.9 ppm)	88%
Cryptophycins	Cryptophycin A*	655.2775 [M+H]⁺	655.2781 [M+H]⁺	$C_{35}H_{43}ClN_2O_8$ (Δ 0.9 ppm)	88%
Acutiphycin	no annotation	-	-	-	-
Aerucyclamides	Aerucyclamide A	535.2156 [M+H]⁺	535,2156 [M+H]⁺	$C_{24}H_{34}N_6O_4S_2$ (Δ 0.0 ppm)	82%
	(3 isomers)	535.2174 [M+H]⁺	535,2156 [M+H]⁺	$C_{24}H_{34}N_6O_4S_2$ (Δ 3.4 ppm)	83%
		535.2161 [M+H]⁺	535,2156 [M+H]⁺	$C_{24}H_{34}N_6O_4S_2$ (Δ 0.9 ppm)	79%
	Aerucyclamide C*	517.2227 [M+H]⁺	517.2228 [M+H]⁺	$C_{24}H_{32}N_6O_5S$ (Δ 0.2 ppm)	83%
	(2 isomers)	517.2224 [M+H]⁺	517.2228 [M+H]⁺	$C_{24}H_{32}N_6O_5S$ (Δ 0.8 ppm)	85%
Anabaenopeptins	Anabaenopeptin A	844.4241 [M+H]⁺	844,4240 [M+H]⁺	$C_{44}H_{57}N_7O_{10}$ (Δ 0.2 ppm)	94%
	Anabaenopeptin C*	809.4542 [M+H]⁺	809.4556 [M+H]⁺	$C_{41}H_{60}N_8O_9$ (Δ 1.7 ppm)	85%
	Anabaenopeptin B	837.4615 [M+H]⁺	837.4617 [M+H]⁺	$C_{41}H_{60}N_{10}O_9$ (Δ 0.3 ppm)	94%
	Anabaenopeptin F*	851.4772 [M+H]⁺	851,4774 [M+H]⁺	$C_{42}H_{62}N_{10}O_9$ (Δ 0.2 ppm)	92%
	Oscillamide Y	858.4399 [M+H]⁺	858.4396 [M+H]⁺	$C_{45}H_{59}N_7O_{10}$ (Δ 0.3 ppm)	79%
Microginins	Microginin SD755*	756.4548 [M+H]⁺	756.4542 [M+H]⁺	$C_{40}H_{61}N_5O_9$ (Δ 0.8 ppm)	32%
	Microginin 742A*	742.4383 [M+H]⁺	742.4386 [M+H]⁺	$C_{39}H_{59}N_5O_9$ (Δ 0.4 ppm)	24%
Cylindrofridins	Cylindrofridin B*	661.3861 [M-H]⁻	661.3876 [M-H]⁻	$C_{38}H_{59}ClO_7$ (Δ 2.3 ppm)	68%
	Cylindrofridin C*	703.3989 [M-H]⁻	703.3982 [M-H]⁻	$C_{40}H_{61}ClO_8$ (Δ 1.0 ppm)	76%
Scyptolin A, B	Scyptolin A*	979.4552 [M-H]⁻	979.4549 [M-H]⁻	$C_{45}H_{69}ClN_8O_{14}$ (Δ 0.3 ppm)	61%

	Scyptolin B*	1120.5383 M-H]$^-$	1120.5339 M-H]$^-$	$C_{52}H_{80}ClN_9O_{16}$ (Δ 3.9 ppm)	56%
Microcystins	MC-LR	995.5557 [M+H]$^+$	995.5560 [M+H]$^+$	$C_{49}H_{74}N_{10}O_{12}$ (Δ 0.3 ppm)	83%
	MC-YR	1045.5353 [M+H]$^+$	1045.5353 [M+H]$^+$	$C_{52}H_{72}N_{10}O_{13}$ (Δ 0.0 ppm)	80%
	[D-Asp3]MC-LR	981.5403 [M+H]$^+$	981.5404 [M+H]$^+$	$C_{48}H_{72}N_{10}O_{12}$ (Δ 0.1 ppm)	72%
	MC-LA	910.4916 [M+H]$^+$	910.4920 [M+H]$^+$	$C_{46}H_{67}N_7O_{12}$ (Δ 0.4 ppm)	87%
	MC-LW	1025.5348 [M+H]$^+$	1025.5342 [M+H]$^+$	$C_{54}H_{70}N_8O_{12}$ (Δ 0.6 ppm)	68%
SMs not known a priori	Cylindrocyclophane D*	667.4236 [M-H]$^-$	667.4215 [M-H]$^-$	$C_{40}H_{60}O_8$ (Δ 3.1 ppm)	77%
	Planktocyclin *	801.4325 [M+H]$^+$	801,4327 [M+H]$^+$	$C_{39}H_{60}N_8O_8S$ (Δ 0.2 ppm)	62%

Table S6. Spectral library matching annotation results obtained for the biomass extracts analyzed in the case study (Table S3). Mass deviation from exact mass in parentheses.

specialized metabolite (annotated)	*m/z* [M+H]$^+$ (accurate mass)	*m/z* [M+H]$^+$ (exact mass)	mol. formula	spectral library matching
Aerucyclamide A	535.2158	535.156	$C_{24}H_{34}N_6O_4S_2$ (Δ 0.4 ppm)	85%
(2 isomers)	535.2160	535.2156	$C_{24}H_{34}N_6O_4S_2$ (Δ 0.7 ppm)	85%
Aerucyclamide B	533.2001	533.1999	$C_{24}H_{32}N_6O_4S_2$ (Δ 0.4 ppm)	90%
Aerucyclamide C	517.2230	517.2228	$C_{24}H_{32}N_6O_5S$ (Δ 0.4 ppm)	95%
Aerucyclamide D	587.1565	587.1563	$C_{26}H_{30}N_6O_4S_3$ (Δ 0.3 ppm)	85%
Anabaenopeptin A	844.4239	844.4240	$C_{44}H_{57}N_7O_{10}$ (Δ 0.1 ppm)	84%
Anabaenopeptin B	837.4619	837.4617	$C_{41}H_{60}N_{10}O_9$ (Δ 0.2 ppm)	98%
Anabaenopeptin F	851.4778	851.4774	$C_{42}H_{62}N_{10}O_9$ (Δ 0.5 ppm)	96%
Oscillamide Y	858.4398	858.4396	$C_{45}H_{59}N_7O_{10}$ (Δ 0.2 ppm)	74%
Oscillapeptin J	1093.4515	1093.4507	$C_{47}H_{68}N_{10}O_{18}S$ (Δ 0.7 ppm)	97%
Cyanopeptolin 1020	1021.5353	1021.5358	$C_{50}H_{72}N_{10}O_{13}$ (Δ 0.0 ppm)	86%
(2 isomers)	1021.5356	1021.5358	$C_{50}H_{72}N_{10}O_{13}$ (Δ 0.2 ppm)	84%
Cyanopeptolin 992	993.5043	993.5040	$C_{48}H_{68}N_{10}O_{13}$ (Δ 0.3 ppm)	84%
Cyanopeptolin B	929.5340	929.5342	$C_{46}H_{72}N_8O_{12}$ (Δ 0.2 ppm)	94%
Cyanopeptolin C	943.5503	943.5499	$C_{47}H_{74}N_8O_{12}$ (Δ 0.4 ppm)	92%
(2 isomers)	943.5501	943.5499	$C_{47}H_{74}N_8O_{12}$ (Δ 0.2 ppm)	81%
Micropeptin HH992	993.5294	993.5292	$C_{50}H_{72}N_8O_{13}$ (Δ 0.2 ppm)	95%
MC-LR	995.5561	995.5560	$C_{49}H_{74}N_{10}O_{12}$ (Δ 0.1 ppm)	89%
MC-YR	1045.5354	1045.5353	$C_{52}H_{72}N_{10}O_{13}$ (Δ 0.1 ppm)	73%
MC-FR*	1029.5407	1029.5404	$C_{52}H_{72}N_{10}O_{12}$ (Δ 0.3 ppm)	76%
[Dha7]MC-LR	981.5405	981.5404	$C_{48}H_{72}N_{10}O_{12}$ (Δ 0.1 ppm)	93%
[D-MeO-Glu6]MC-YR	1059.5511	1059.5511	$C_{53}H_{74}N_{10}O_{13}$ (Δ 0.1 ppm)	87%
[D-Asp3,(E)-Dhb7]MC-RR	1024.5576	1024.5574	$C_{48}H_{73}N_{13}O_{12}$ (Δ 0.2 ppm)	77%

MC-HilR	1009.5723	1009.5717	$C_{50}H_{76}N_{10}O_{12}$ (Δ 0.6 ppm)	89%
MC-LA	910.4927	910.4920	$C_{46}H_{67}N_{7}O_{12}$ (Δ 0.8 ppm)	94%
MC-LF	986.5237	986.5233	$C_{52}H_{71}N_{7}O_{12}$ (Δ 0.4 ppm)	92%
Planktocyclin	801.4331	801.4327	$C_{39}H_{60}N_{8}O_{8}S$ (Δ 0.5 ppm)	83%
